# Notum enhances gastric cancer stem-like cell properties through upregulation of Sox2 by PI3K/AKT signaling pathway

**DOI:** 10.1007/s13402-023-00875-w

**Published:** 2023-09-26

**Authors:** Yi Liu, Hui Chen, Lanshu Xiao, Ping Dong, Yanhui Ma, Yunlan Zhou, Junyao Yang, Bingxian Bian, Guohua Xie, Lei Chen, Lisong Shen

**Affiliations:** 1 Department of Clinical Laboratory, Xinhua Hospital, Shanghai Jiao Tong University School of Medicine, Shanghai, 200092 China; 2 Department of General Surgery, Xinhua Hospital, Shanghai Jiao Tong University School of Medicine, Shanghai, 200092 China; 3 Institute of Artificial Intelligence Medicine, Shanghai Academy of Experimental Medicine, Shanghai, 200240 China; 4 Faculty of Medical Laboratory Science, College of Health Science and Technology, Shanghai Jiao Tong University School of Medicine, Shanghai, 200092 China

**Keywords:** Notum, Early-stage gastric cancer, Stemness features, Sox2, PI3K/AKT signaling pathway, Caffeine

## Abstract

**Purpose:**

Considerable evidence suggests that tumor cells with stemness features contribute to initiation, progression, recurrence of gastric cancer (GC) and resistance to therapy, but involvement of underlying regulators and mechanisms remain largely unclear. However, the clinical significance and biological function of Notum in GC tumor sphere formation and tumorigenesis remain unclear.

**Methods:**

Bioinformatics analysis, RT-qPCR, western blot and imunohistochemistry staining were applied to characterize Notum expression in GC specimens. The early diagnostic value of Notum was analyzed by logistic regression analysis method. Cancer stemness assays were used in Notum knockdown and overexpressing cells in vitro and in vivo. RNA-seq was employed to reveal the downstream effectors of Notum.

**Results:**

Notum is highly expressed in early stage of GC patients and stem-like GC cells. For discriminating the early-stage and advanced GC patients, the joint analysis had a better diagnostic value. Overexpression of Notum markedly increased stemness features of GC cells to promote tumor sphere formation and tumorigenesis. Conversely, Notum knockdown attenuated the stem-like cell properties in vitro and in vivo. Mechanically, Notum upregulates Sox2 through activating the PI3K/AKT signaling pathway. Notum inhibitor Caffeine exhibited a potent inhibitory effect on stemness features by impairing the PI3K/AKT signaling pathway activity and targeting Sox2.

**Conclusion:**

Our findings confer a comprehensive and mechanistic function of Notum in GC tumor sphere formation and tumorigenesis that may provide a novel and promising target for early diagnosis and clinical therapy of GC.

**Supplementary Information:**

The online version contains supplementary material available at 10.1007/s13402-023-00875-w.

## Background

Gastric cancer (GC) remains one of the most incident neoplasms and the third leading cause of cancer-related death [1].The dysregulation of oncogenic and tumor suppressor genes and pathways result in the stepwise accumulation of numerous epigenetic alterations [2]. It has been reported that gastric cancer stem cells (GCSCs) function as vital contributors to tumor initiation, progression, metastasis and relapse, drug resistance [3].GCSCs possess the ability of unlimited self-renewal and heterogeneous differentiation, and they have been described highly resistant to currently available chemotherapy and targeted therapy in GC [4, 5]. Therefore, understanding the molecular mechanisms that enhances the stemness of GC cells will provide new strategies to improve GC treatments.

Notum (Wingful), a palmitoleoyl-protein carboxylesterase, is a secreted antagonist of Wingless (Wg, *Drosophila* Wnt1), encoding an α/β-hydrolase enzyme with similarity to pectin acetylesterases from plants [6–8]. It is reported that Notum influences Wingless protein distribution by modifying cell surface proteoglycans and is induced by high levels of Wingless signaling at the same time [6]. Initial studies in fruit flies suggested that Notum was able to cleave the link between the membrane and glypicans (GPCs) which complex with Wnt [9].In planarians, Notum acts as a target and inhibitor of Wnt signaling pathway, controlling the switch-like behavior of the head-versus-tail regeneration decision [10]. That is to say, the feedback regulation between Wnt and Notum, respectively expressing at opposite brain poles, plays an important role in development of tissues and organs [11].In zebrafish, Notum develops a self-regulatory loop that restricts Wnt signaling pathway to modulate vertebrate development [12, 13]. Recent studies emerged to indicate that Notum might be involved in progression of colorectal cancer (CRC), neonatal respiratory disease, oral squamous cell carcinoma (OSCC), and aberrantly expressed Notum was regulated by β-catenin/TCF, suggesting Notum functions as a potential new biomarker in CRC [14–18]. In addition, Hua Wang et al. identified Notum enriched for CRC cancer stem cells with single-cell analysis, and it was expressed at remarkably higher levels in cancer stem cells distinct from any other type [19]. Another study also showed that Notum was specially expressed in slow-cycling LGR5^+^ cells from xenografted human colon tumors and was upregulated in colon cancer clinical specimens [20]. Notum inhibitors have been explored, and the inhibition of Notum shows promise for cancer therapy [21]. However, the role of Notum in GC tumor sphere formation and tumorigenesis and its mechanism remains unknown.

Here, to confirm the oncogenic role Notum in GC tumor sphere formation, tumorigenesis, and the therapeutic potential of Notum inhibition, we performed a series of functional experiments in vitro and in vivo. Our study showed that Notum was overexpressed in GC, especially in early-stage GC patients and stem-like gastric cancer cells. Diagnostic power of the combined panel outperformed that of single tumor marker for establishing a diagnosis of early-stage GC. Furthermore, Notum promoted tumor sphere formation and tumorigenesis through upregulation of Sox2 by activating the PI3K/AKT signaling pathway. Notum inhibitor Caffeine exhibited a potent inhibitory effect on tumor sphere formation, suggesting a possible application for clinical therapy.

## Materials and methods

### Human GC samples

Gastric tumor tissues and serum were obtained from GC patients who underwent curative surgery at Xinhua Hospital, Shanghai Jiao Tong University School of Medicine, China, between 2009 and 2019 (Additional file 2:Table S1 and Table S3). The tissues and serum were stored at -80 °C and subjected to mRNA and protein extraction for reverse transcription quantitative real-time PCR (RT-PCR) and western blot. The GC tissues for IHC containing 12 normal samples and 63 tumors were purchased from Wuhan Servicebio Technology (Additional file 2: Table S2). All patients were diagnosed by pathological analyses based on the TNM criteria defined by the International Union Against Cancer (UICC). The study protocol conformed to the ethical guidelines of the Declaration of Helsinki and was approved by the Institutional Review Board and Ethics Committee of Xinhua Hospital.

### Serum concentrations of Notum and tumor markers

The serum concentrations of AFP, CEA, CA199, CA125, CA724, CA211, CA242, CA153 and NSE were detected by the Roche Cobas e801 automatic-electro-chemiluminescence immunoassay analyzer. The reagents, standards and controls were purchased from Roche. The serum levels of SCC and CA50 were detected by the Abbott I1200 and Maglumi 400 respectively. Serum Notum was determined by the Elisa assay as the following protocol. 100µL of serum was transferred from each sample to 96-well plate coated with anti-Notum antibody. After mixing, incubation and washing, the levels were calculated according to the standard curve.

### Cell lines and culture

The human GC cell lines (SGC-7901, MGC-803, BGC-823, and MKN-45) and human embryonic stem(hES) cell line H9 were purchased from the Chinese Academy of Sciences Cell Bank of Type Culture Collection (Shanghai, China) and Quicell Bioscience, Inc. (Shanghai, china). GC cell lines were cultured at 37 °C in anatmosphere containing 5% CO_2_ in Dulbecco’s Modified Eagle’s Medium (DMEM, HyClone, USA)supplemented with10% fetal bovine serum (FBS, Gibco, USA), 100 U/ml penicillin and 100 µg/ml streptomycin (Gibco, USA).H9 cell line was cultured at 37 °C in an atmosphere containing 5% CO_2_ in Advanced DMEM/F12 supplemented with knockout serum replacement (20%), non-essential amino acids (1×), L-glutamine (1×), penicillin/streptomycin (1×), β-mercaptoethanol (1×) and FGF-2 (4 ng/ml) (Peprotech, USA).For tumor sphere cultures, cells were seeded in dishes precoated with 18 mg/ml polyHEMA (Sigma-Aldrich, USA) and cultured in serum-free DMEM/F12 media(Gibco, USA) supplemented with 20 ng/ml EGF and 10 ng/ml bFGF (Peprotech, USA), 1% N2 and 2% B27. The mediums and most supplements were purchased from the Gibco in USA. LY294002 and 740Y-P (Selleck, USA), Caffeine (APEXBIO, USA), Melatonin (Selleck, USA), ABC99 and LP-922,056 (Glpbio, USA), Lipofectamine^TM^3000 Transfection Reagent (ThermoFisher, USA) were used in this study.

### Lentivirus production and transfection

Lentiviral particles carrying the human Notum coding sequence, Sox2 short hairpin RNA (shSox2) and their respective controls were constructed by Genomeditech, Shanghai, China. MKN-28 and MGC-803 cells were infected with lentiviral particles, and the expression of Notum and Sox2 were measured using RT-qPCR and western blot. For Crispr/Cas9 knockout experiment, the guide RNA targeting sequences of Notum and Cas9 Lentiviral vectors were designed and synthesized by Genomeditech. The sgRNAs and Cas9 vectors were transfected in MKN-45 and BGC-823 cells, the single clone was isolated and expanded, and western blot was used to select the clones according to the expression of Notum.

### Cell viability and adhesion-dependent colony formation assay

GC cells were seeded in a 96-well plate at 1500–3000 cells per well for 0–6 days, and cell viability was detected with 3-(4,5-dimethyl-2-thiazolyl)-2,5-diphenyl-2-H-tetrazolium bromide (MTT) (Sigma-Aldrich, USA). The optical density at 490 nm was measured in a multiwell plate reader (FLX800, Bio-TEK, USA). GC cells were plated in 60-mm dishes at a density of 2 × 10^3^ cells per well for the adhesion-dependent colony formation assay. The culture medium was changed every 3–4 days. Then, 3–4 weeks later, the remaining colonies were fixed with 4% paraformaldehyde and stained with crystal violet. The colonies were counted according to the defined colony size.

### RNA extraction and quantitative real-time PCR

Total RNA was extracted using TRIzol reagent (Invitrogen, USA) according to the manufacturer’s instructions. The concentration and quality of the total RNA were assessed with a NanoDrop spectrophotometer (Thermo Fisher Scientific, USA). For mRNA expression analysis, reverse transcription was performed using PrimeScript RT master mix (TaKaRa, Japan). Quantitative real-time PCR analysis was performed in triplicate on a 7900 HT real-time PCR system (Applied Biosystems, USA) using SYBR Premix Ex Taq (TaKaRa, Japan), and the expression level of Actin was used as an endogenous control. The results were analyzed using the 2^–ΔΔct^ calculation method. The primers used for the experiments are listed in Additional file 2:Table S4.

### Immunohistochemical staining

Specimens were prepared as previously described [22]. Automated image acquisition was performed using an AperioScanScope XT slide scanner system with a 20×objective (Aperio Technologies).

### Western blot analysis

The cells were lysed in equal volumes of ice-cold lysis buffer with aprotease inhibitor cocktail. Cell lysates were separated by SDS-PAGE and then transferred to a 0.2-µm PVDF membrane (Bio-Rad, USA). After blocking with Odyssey blocking buffer (Li-COR Biosciences, USA), the membrane was incubated with primary antibody (1:1000) at 4 °C overnight, followed by incubation with IRDye 800CW or 680 secondary antibodies (1:5000, LI-COR Biosciences, USA). Actin was used as an endogenous control. An Odyssey infraredmaging system was used to visualize the targeted protein bands. All antibodies used in the experiments are listed in Additional file 2: Table S5.

### Immunofluorescence assay

Specimens were prepared as previously described. Images were captured using a Leica SP5 laser scanning confocal microscope [22].

### Flow cytometry

GC cells were trypsinized and washed twice in 1×PBS, after being resuspended in 100 µl FBS, fluorochrome conjugated antibodies against CD44, CD54, CD133, CD326 and their respective isotype controls were added to stain for 30 min at 4 °C. Following being washed twice in 1×PBS, labeled cells were analyzed by flow cytometry on a FACS CantoII flow cytometer (BD Biosciences) and the results were analyzed with FlowJo software (Tree Star).

### In vitro limiting dilution assay

GC cells were seeded into 96-welldishes precoated with polyHEMA at cell doses and incubated in spheroid-forming conditions for 14 days. Colony formation was assessed by visual inspection. Based on the frequency of wells with spheroids, the proportion of spheroid-initiating cells was counted and analyzed.

### In vivo limiting dilution assay

4-6-week-old male nude mice were purchased from the Shanghai Laboratory Animal Center of China (Shanghai,China) and received humane care. Various amounts of GC cells (1 × 10^6^, 1 × 10^5^ and 1 × 10^4^) were injected subcutaneously (n = 3 per group). Mice were monitored for five weeks, sacrificed and tumor nodules were counted. All animal procedures were carried out with the approval of the Institutional Committee of Shanghai Jiao Tong University School of Medicine for Animal Research.

### In vivo secondary xenograft experiment

Treated or untreated GC cells (1 × 10^6^ cells in 200 µl of 1×PBS) were subcutaneously injected into the right flanks of nude mice to establish tumors. After five weeks, mice were sacrificed, and tumors were isolated. Primary tumors were separated into the single cell suspension and injected into the secondary mice to establish tumors.

### RNA sequencing

Treated or untreated GC cells (1 × 10^6^ cells) were collected, and total RNA was extracted. All samples were sent to BGI Corporation (Shenzhen, China) for further RNA sequencing detection and analysis via BGISEQ-500 sequencer. The differentially expressed genes (DEGs) were analyzed by R packages and, the criteria were defined as a P value < 0.05 and fold change > 2.0 or fold change < − 2.0. The analysis of pathways and gene functions were performed based on the Kyoto Encyclopedia of Genes and Genomes (KEGG) and Gene Ontology (GO).

### Bioinformatics analysis

Four public GC microarray gene profiling datasets (GSE112369, GSE33874, GSE36563 and GSE7181) were downloaded from the Gene Expression Omnibus (GEO) on the NCBI web server. The expression data of normal stomach tissue and tumor stomach tissues were obtained from The Cancer Genome Atlas (TCGA), and Weighted correlation network analysis (WGCNA) was conducted. To screen the DEGs between GC and normal samples, the “limma” package of R was performed and the differential expression of mRNAs were visualized with the “pheatmap” of R [23, 24]. STRING 10, a search tool for the retrieval of interacting genes/proteins (http://string-db.org/), was used to construct the protein-protein interaction (PPI) between proteins encoded by differentially expressed genes. The prognostic significance of Notum in gastric cancer patients was analyzed by data mining in the KM-Plotter plotter database(http://kmplot.com). R packages and cytoscape 3.7.1 were used to analyze the DEGs, KEGG, GO, network analysis and visualization.

### Statistical analysis

Statistical significance between groups was determined by two-tailed Student’s t-test and one-way ANOVA. Differences were significant when P < 0.05. All statistical data are shown as the means ± standard deviations (SDs) and were analyzed for statistical significance with GraphPad Prism 5.0 for Windows (GraphPad Software, USA). The diagnostic value was evaluated by the area under the curve (AUC) of the receiver operator characteristic (ROC) curve, the cutoff value was determined by the Youden index. The combinations of the indicators were analyzed by the logistic regression analysis method.

## Results

### The pattern and significance of Notum expression in GC

**Fig. 1:**
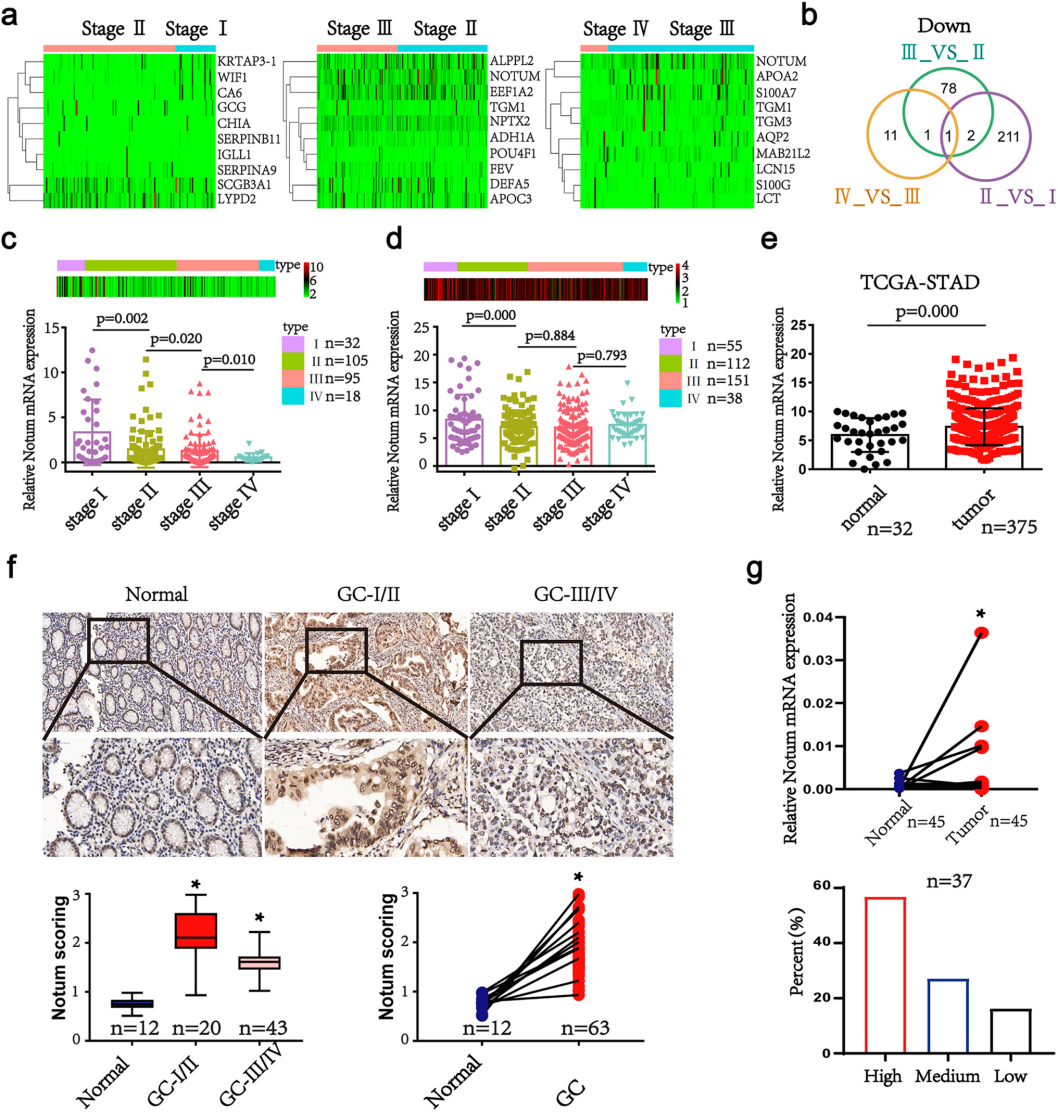
Notum is upregulated in GC tissues and early stage of GC patients. **a**, **b** Hierarchical clustering analysis of the top 10 genes of the significantly (> 2.0-fold) upregulated or downregulated mRNAs in different stages of GC patients (a) and graphical representation of the overlapped downregulated gene (1 gene) among three groups (Stage II VS Stage I, Stage III VS Stage II and Stage IV VS Stage III) (b). **c**, **d** Notum levels in GC patients with different tumor grades in TCGA dataset from RNA-seq data (c) and mRNA microarray data (d). **e** Notum mRNA expression in TCGA datasets in GC tissues compared to normal specimens. **f** Representative images of Notum immunohistochemical (IHC) staining in normal and GC tissues (upper). IHC scores of normal and GC tissues based on Notum expression analyzed by IHC staining (lower). **g** The expression of Notum mRNA (45 pairs) (upper) and protein (37 pairs) (lower) detected by RT-qPCR and western blot in GC tissues

In an attempt to identify oncogenes associated with initiation and progression of GC, we first analyzed the mRNAs profiles of different stages of GC for whom clinical data were available in GC data set of TCGA. We found that Notum amplification was associated with the GC progress, which was highly expressed in GC tissues, especially in early stage of GC patients (Fig. 1a and e). Compared with other cancers, Notum was aberrantly upregulated in GC, suggesting Notum may play a potentially crucial role in initiation and progression of GC (Additional file 1: Fig. S1A and S1B). To confirm this finding, we investigated Notum immunostaining of tissue slides containing 63 GC specimens and 12 normal tissues. The results showed that Notum immunostaining was stronger in I and II stage of GC tissues versus III and IV stage of GC tissues and normal stomach tissues (Fig. 1f). Moreover, we verified the higher expression of Notum in GC specimens and adjacent tissues by qRT-PCR (n = 45) and western blot (n = 37) (Fig. 1g, Additional file 1: Fig. S1C). However, there was no difference of survival time between patients stratified by a median cutoff of Notum expression with higher Notum expression (> median) and those expressing lower levels of Notum (≤ median) (Additional file 1: Fig. S1D). Collectively, our results indicated that Notum might be a critical oncogene to promote initiation and progression of GC.

### Early diagnostic value of the concentrations of serum Notum and tumor markers for GC

**Fig. 2:**
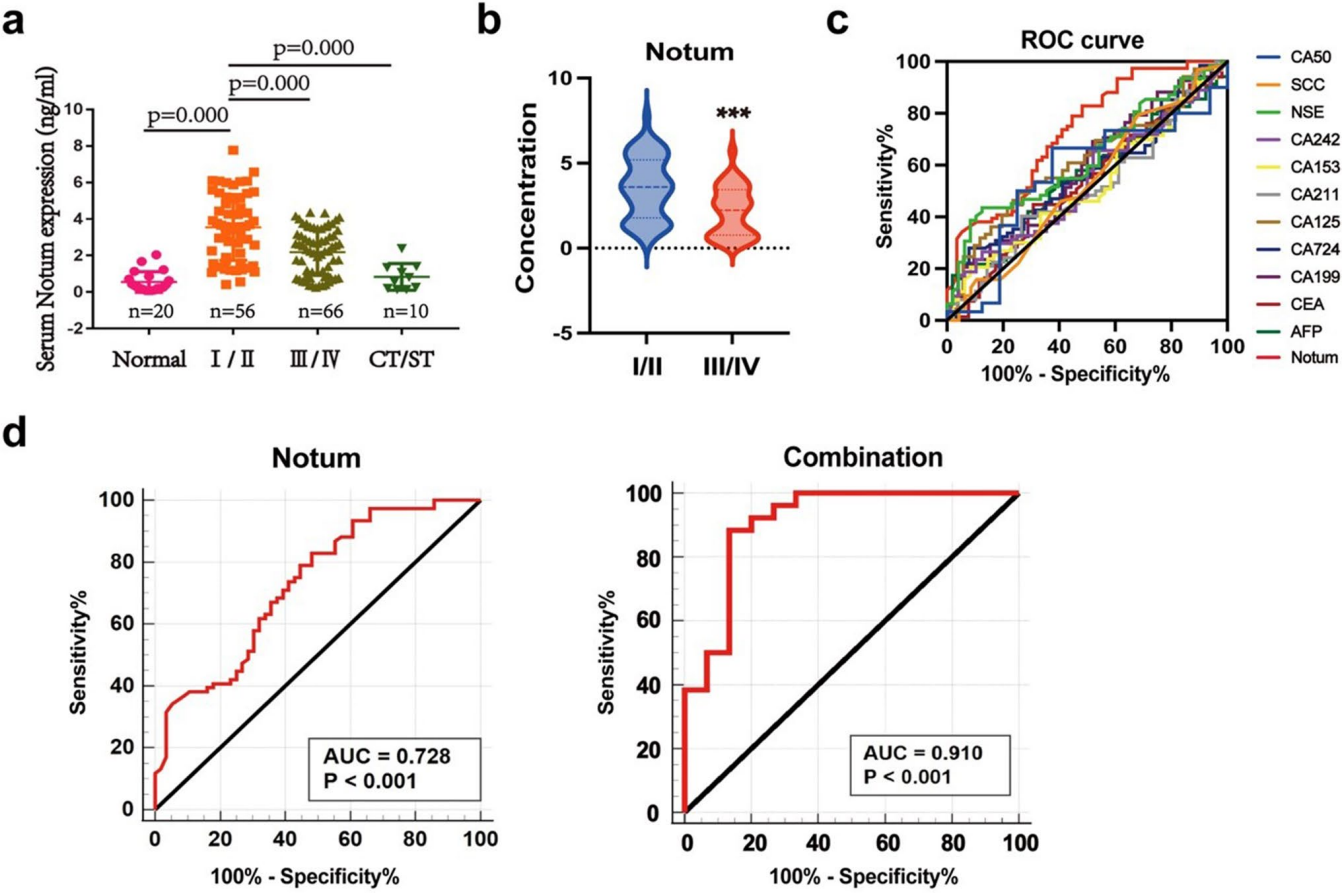
Diagnostic value of Notum and tumor markers for discriminating the early-stage GC patients and advanced patients. **a** Measurement of serum Notum levels in different stages of GC patients, normal samples, and patients after chemotherapy (CT) and surgical therapy (ST). **b** Serum levels in early-stage GC patients and advanced patients. **c** Receiver operating characteristic curves for the serum Notum and tumor markers to discriminate early-stage GC patients from advanced patients. **d** Comparison of early diagnostic value of Notum and joints analysis

As shown in Fig. 2a, the concentration of serum Notum in GC patients was higher than Normal group and patients after chemotherapy (CT) and surgical therapy (ST). Especially, serum Notum was significantly higher in early-stage GC patients than advanced patients (Fig. 2b). Meanwhile, serum levels of AFP, CEA, CA199, CA724, CA153, CA211, CA242, CA125, SCC and CA50 failed to distinguish early-stage GC patients from advanced patients, except for NSE (Additional file 1: Fig. S2). We next assessed the diagnostic capacity of Notum and each tumor marker by computing their ROC curve. Among these tumor markers, Notum possessed the highest diagnostic power in discriminating early-stage GC patients and advanced patients with AUC at 0.728 (sensitivity = 82.89%, specificity = 51.79%) (Fig. 2c and d, Additional file 1: Fig. S3A and Additional file 2: Table S6). Further analysis suggested that the most powerful diagnostic panel combination of Notum and other 11 tumor markers received an AUC of 0.910, with sensitivity of 88.46% and specificity of 86.67%, which was higher than the combination of 11 tumor markers, with an AUC of 0.839 (Fig. 2d, Additional file 1: Fig. S3B). Moreover, significant synergistic effect was also found in the combination of Notum and AFP, which may be due to the high specificity of AFP compensating the low specificity of Notum. However, there was no significance differences among the combination of Notum and other traditional biomarkers (Additional file 1: Fig. S3A and Additional file 2: Table S6). Our results indicated that Notum could serve as a potential predictive marker for the diagnosis of early-stage GC.

### GCSCs enrichment after cisplatin treatment

Given that GCSCs are resistant to chemotherapeutic drugs and suspension culture has been used to expand GCSCs, we attempted to enrich for GCSCs by culturing GC cell lines treated with chemotherapeutic drugs in serum-free medium under low-adherent conditions (Additional file 1: Fig. S4A). To confirm the stemness characteristics of spheroid forming cells exposure to chemotherapeutic drugs, a series of cancer stemness assays were conducted, including stem cell-associated markers and genes, limiting dilution assay in vitro and in vivo, single-cell tumor sphere formation in vitro and secondary transplantation in vivo. As shown in Additional file 1: Fig. S4B and S4C, after treatment with 5-FU and cisplatin (Cis), the sphere cells highly expressed stem-cell markers and genes. In addition, the sphere cells were enzymatically dissociated into single cells and re-cultured to generate sub-spheroid bodies again, and the results revealed that body-forming efficiency increased in the sphere cells treated with 5-FU and Cis (Additional file 1: Fig. S4D). The tumorigenicity of GCSCs was examined by the secondary transplantation experiment and limiting dilution assay in immunocompromised nude mice. Inoculations of 10^6^ tumor cells isolated from the primary tumors developed by 10^6^ sphere cells treated with Cis or sphere cells were able to develop tumors in 5/5 and 1/5 of mice, respectively (Additional file 1: Fig. S4E). And the tumor weights were recorded to highlight the effect of Notum knockdown on tumor development (Additional file 1: Fig. S4F). Moreover, inoculation with as few as 10^4^ sphere cells treated with Cis resulted in the formation of tumors in all three mice, whereas only one tumor was found after 10^4^ sphere cells were administered and the tumor weights were also compared (Additional file 1: Fig. S4G and S4H). Therefore, Cis-treated sphere cells may largely enrich the stem-like cells and exhibit the stemness properties much closer to GCSCs.

### Notum may involve in GCSCs development

To elucidate the role of Notum in GC pathogenesis, we carried out gene set enrichment analysis (GSEA) using public datasets (GSE65801), and the results showed that high Notum expression was positively associated with Wnt/β-catenin and PI3K/AKT signaling pathways, which was further confirmed by analysis of The Cancer Genome Atlas (TCGA) database (Additional file 1: Fig. S5A-5D). Additionally, the correlation analysis of String indicated that Notum was significantly associated with genes involved in cancer stem cell development, such as FGFs family, implying a pivotal role of Notum in GCSCs (Additional file 1: Fig. S5E).

**Fig. 3:**
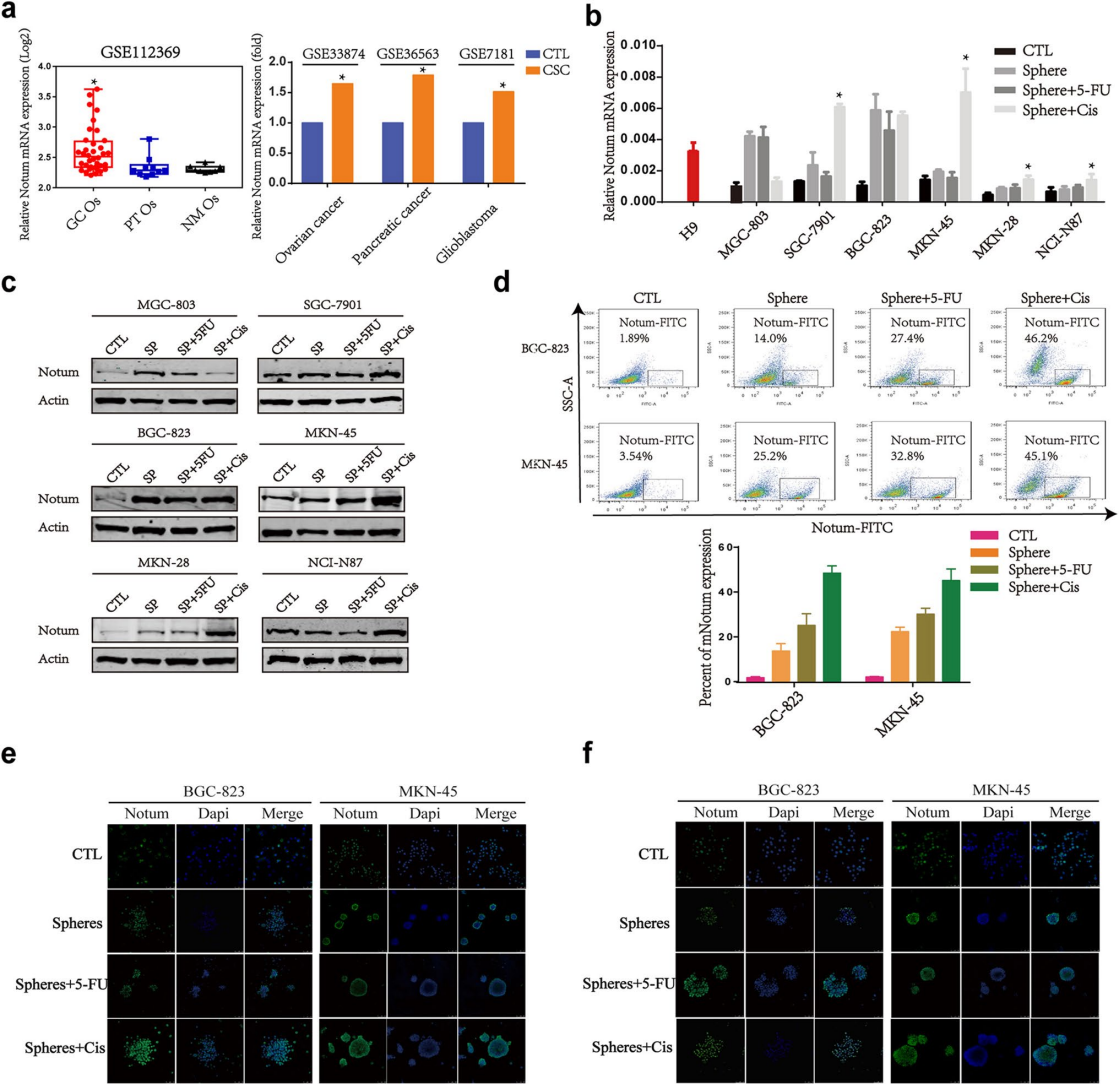
Notum is upregulated in GCSCs. **a** Notum levels in CSCs of GC and other cancer from GEO datasets. GC Os, gastric cancer organoids; PT Os, para-carcinoma tissue organoids; NM Os, normal matched organoids. **b, c** RT-qPCR (b) and western blot (c) analysis of Notum levels in tumor spheres and spheres treated with chemotherapeutic drugs including 5-FU and Cisplatin (Cis). H9 cells, the human embryonic stem cells, as the positive control. d Flow cytometric analysis for CD44 cell surface expression in 2 GC cell lines cultured as spheres. e, f The representative images of immunofluorescence staining of membrane Notum (e) and intracellular Notum (f) in tumor spheres generated by BGC-823 and MKN-45 cell lines

Since Notum may involve in the development of GCSCs, we wonder whether Notum is upregulated in GCSCs and characterized as the stem marker. To explore this, the mRNA microarray data from the NCBI’s Gene Expression Omnibus (GEO) database were deposited to analyze Notum levels in CSCs. As shown in Fig. 3a, Notum was highly expressed in gastric cancer organoids (GC Os) as well as in other cancers. Then, we detected Notum levels in GC cell lines, sphere cells, 5-FU treated sphere cells and Cis-treated sphere cells and found a gradual increase of the Notum mRNA and protein levels (Fig. 3b and c). Besides, flow cytometric analysis (FCA) of Notum levels in cell membrane suggested that Notum + subpopulations could be isolated and much more enriched in spheres cells treated with chemotherapeutic drugs, especially with Cisplatin (Fig. 3d). The distribution of Notum in GC cells was identified, and the results showed that Notum was not only highly expressed in membrane but also cytoplasm and nucleus and manifested the highest expression in sphere cells treated with Cisplatin (Fig. 3e and f).

### Notum enhances tumor sphere formation and tumorigenicity

**Fig. 4:**
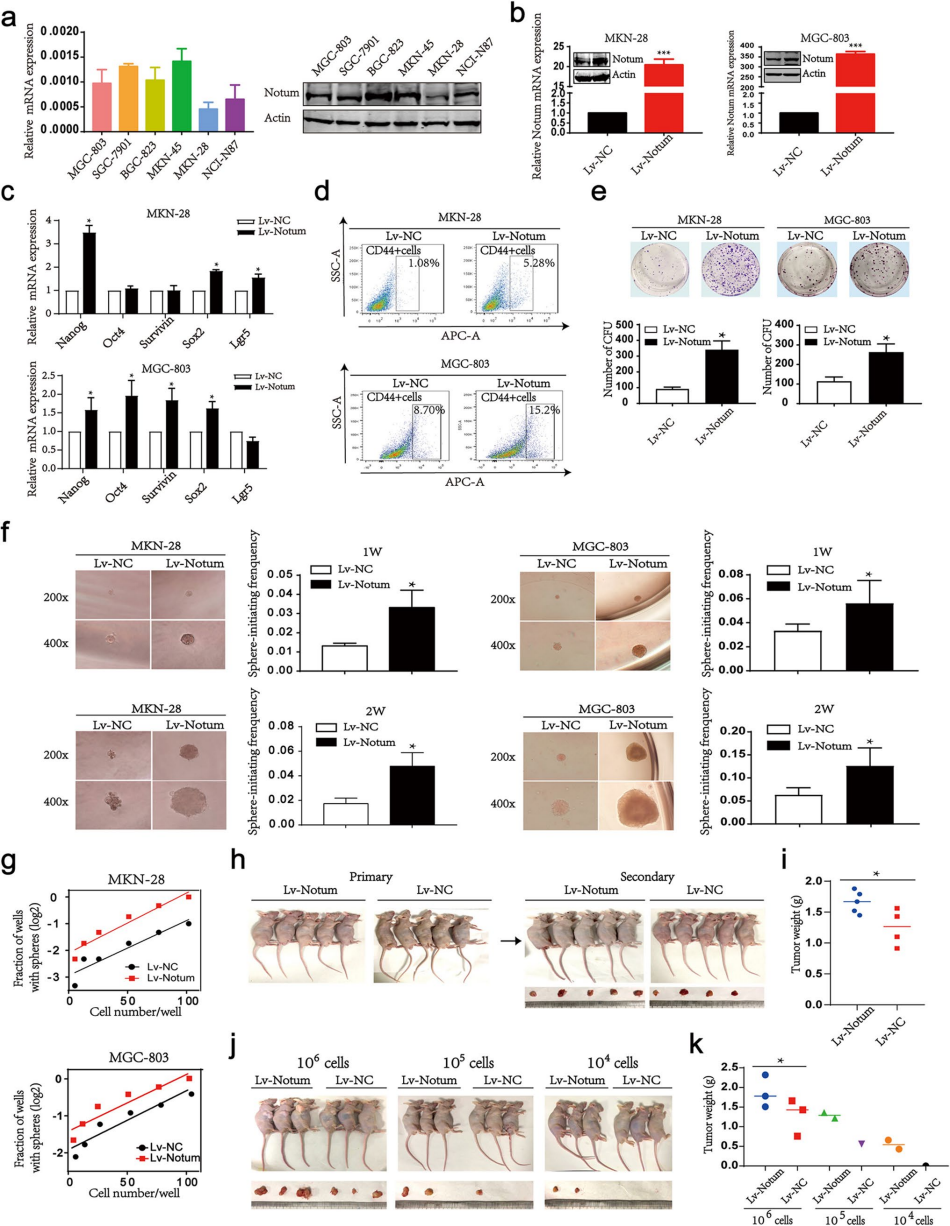
Overexpression of Notum promotes GC cell stemness and tumorigenesis in vitro and in vivo. **a** The expression of Notum mRNA (left) and protein (right) was detected by RT-qPCR and western blot, respectively, in 6 GC cell lines. **b** The overexpression efficiency is confirmed by RT-qPCR and western blot in MKN-28 and MGC-803 cells transduced with Notum lentivirus (Lv-Notum) or control (Lv-NC) individually. **c** The RT-qPCR analysis of the relative mRNA levels for stemness genes, including Survivin, Lgr5, Sox2, Nanog and Oct4. **d** Flow cytometric analysis for CD44 cell surface expression in MKN-28 and MGC-803 cells transduced with Lv-NC or Lv-Notum vectors. **e** Colony formation assay in control and Notum-overexpressing MKN-28 and MGC-803 cells. **f** Sphere formation abilities of Notum-overexpressing MKN-28 and MGC-803 cells. Representative images (left) and the sphere-formation efficiencies (right) are shown. **g**, **j**, **k** Limiting dilution assays are performed in vitro (g) and in vivo (j, k). **h**, **i** The secondary xenograft transplantation experiment in vivo is applied to detect the tumorigenesis of Notum-expressing MGC-803 cells (h) and the statistical analysis of tumor weights is performed (i)

To address whether Notum played a key role in GC stemness, we analyzed the Notum levels in six GC cell lines, and applied lentivirus delivery of Notum to achieve ectopic Notum overexpression in two GC cell lines (Fig. 4a and b). Most stemness genes were significantly up-regulated by exogenous Notum expression (Fig. 4c). Subsequently, FCA showed that enforced Notum expression could lead to an increase in proportion of cells expressing CD44 (Fig. 4d).

Key properties of GCSCs are efficient spheroid formation and enhanced tumorigenicity. Therefore, we first inspected the efficiency of colony formation in vitro and found that overexpression of Notum levels could significantly promote the colony formation (Fig. 4e). Next, suspension-cultured cells with Notum overexpression had more and larger tumor spheroids than controls (Fig. 4f). Consistently, we applied in vitro and in vivo limiting dilution assays to confirm the expansion of GCSCs by Notum. The limiting dilution analysis revealed increase in sphere-initiating cells by enforced Notum in GC cells (Fig. 4g, j and k). Specifically, cells isolated from primary tumors were used for secondary transplantation, and after inoculation of GC cells dissected from the first-generation mice into the second-generation mice, tumors were found to be more and larger promoted by Notum overexpression (Fig. 4h and i). In sum, these results supported the concept that Notum might play a pivotal role in tumor sphere formation and tumorigenicity.

### Notum is required for maintaining GCSCs stemness

**Fig. 5:**
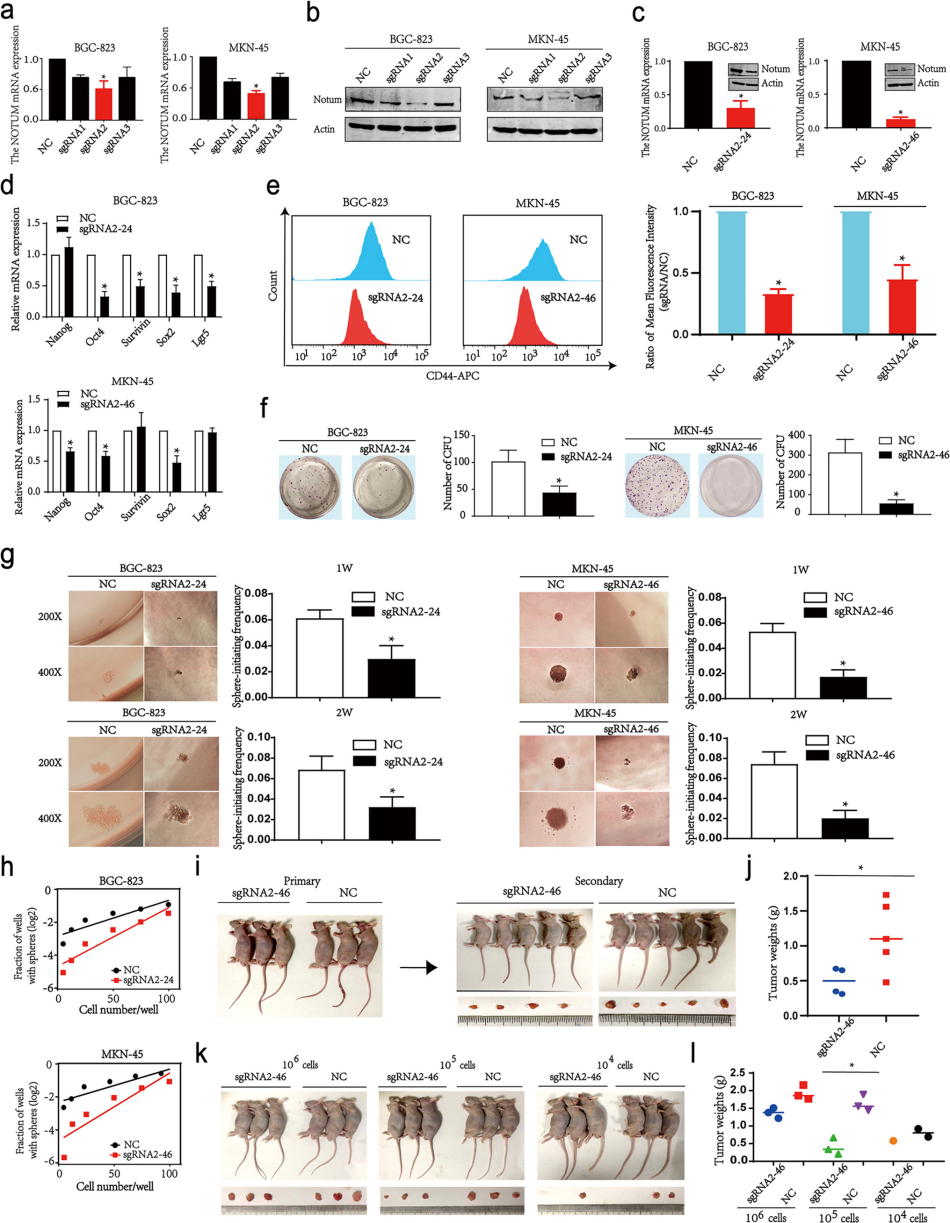
Notum modulates stemness and tumorigenesis ability in GC cell lines. **a**, **b**, **c** The silencing efficiency of Notum with Crispr/Cas9 system in BGC-823 and MKN-45 cells was confirmed by RT-qPCR and western blot. **d** The relative expression levels of Nanog, Oct4, Survivin, Sox2, and Lgr5 mRNAs were quantified by RT-qPCR. **e** Flow cytometric analysis of CD44 cell surface marker in BGC-823 and MKN-45 cell lines after silencing of Notum. **f** The effect of Notum expression on the capacity of colony formation in GC cell lines. **g** Representative images and a statistical histogram showing the decreased numbers and volumes of spheres formed by GC cells transduced with Crispr/Cas9 vectors compared with the negative controls. **h**, **k**, **l** The limiting dilution assays in vitro (h) and in vivo (k, l) to test the frequency of sphere-initiating cells. **i**, **j** Secondary xenograft experiment in MKN-45 cells with reduced Notum to assess the GCSCs frequency

On the contrary of overexpression, we used the Crispr/Cas9 system to knock down the expression of Notum in BGC-823 and MKN-45 cell lines (Additional file 1: Fig. S6A-6 C, Fig. 5a, b and c). After Notum inhibition, stemness genes were significantly suppressed (Fig. 5d). A reduction in Notum level lowered the proportion of GCSCs that could be labeled by CD44 (Fig. 5e). Importantly, knockdown of Notum dramatically impaired the colony formation and sphere-formation efficiency of GC cells (Fig. 5f and g). Limiting diluting assays showed that spheroids-initiating ability was significantly inhibited by Notum knockdown, both in vitro and in vivo (Fig. 5h, k and l). And Notum knockdown retarded tumor growth, as observed in tumors removed from the second-generation mice (Fig. 5i and j). Taken together, our data suggested that Notum is required for maintaining GCSCs stemness.

### Notum activates the PI3K/AKT signaling pathway and upregulated Sox2 expression

**Fig. 6:**
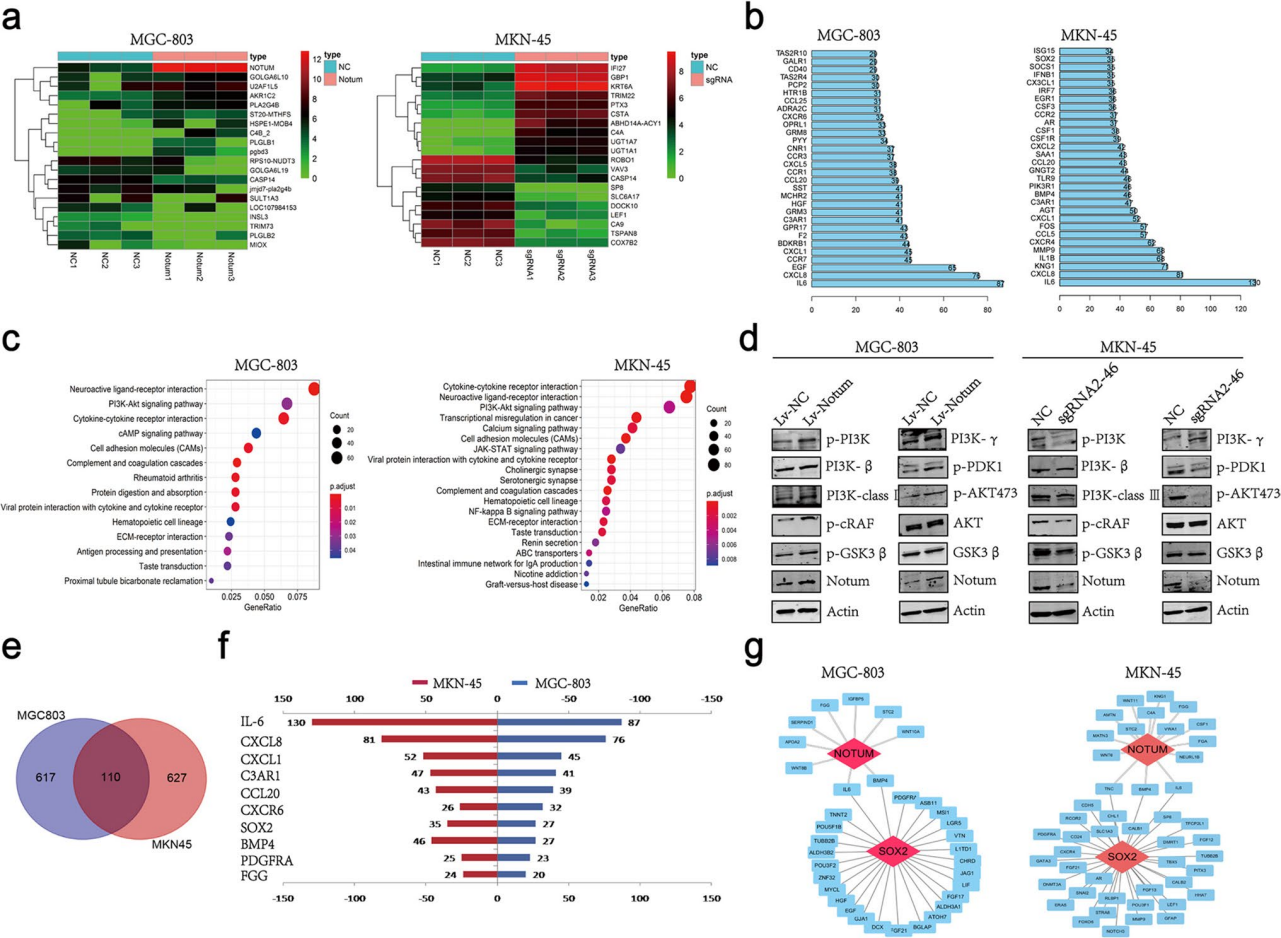
Notum activates PI3K/AKT signaling pathway. **a** Hierarchical clustering of the top 20 transcripts that were altered (≥ 2-fold change, p < 0.05) in MGC-803 and MKN-45 cell lines. **b** Histogram of the top 30 genes analyzed by String analysis of altered transcripts with upregulated or downregulated expression (combined score > 0.4). **c** KEGG analysis of altered transcripts with upregulated or downregulated expression. **d** The protein levels of the PI3K/AKT signaling pathway were confirmed by western blot in MGC-803 cells with Notum overexpression vectors and MKN-45 cells with Notum silencing vectors. **e, f** Venn diagram and histogram show the cross genes analyzed by String (e) and the top 10 genes are shown (f). **g** Visualization analysis of the correlation between Notum and Sox2 with Cytoscape software in MGC-803 and MKN-45 cell lines

To identify critical pathways and genes which mediate the effect of Notum on GCSCs, we compared the expression profiles of Notum overexpression and knockdown. Unsupervised hierarchical clustering and analysis of variance-based statistical analysis identified the differentially expressed genes (DEG) whose foldchange was ≥ 2, and the top 20 were listed (Fig. 6a). Subsequently, the correlation analysis of the key gene expression was carried out to confirm the relevance within the DEGs (Fig. 6b). Regarding gene and signaling pathway enrichment, the Gene Ontology (GO) and KytoEncyopeida of Genes and Genomes (KEGG) analysis were conducted, and we focused our attention on the PI3K/AKT signaling pathway (Fig. 6c, Additional file 1: Fig. S7A and 7B). Using western blot, we observed that p-PI3K, p-AKT (Thr473) and p-GSK3β levels were significantly increased in MGC-803 cells transduced with exogenous Notum but reduced in response to the presence of Notum-Crispr/Cas9 vector in MKN-45 cells (Fig. 6d). Further study based on the correlation analysis suggested that 110 node genes (Pearson correlation > 0.4) were co-expressed in both MGC-803 and MKN-45 cells (Fig. 6e). Notably, Sox2 had 27 node numbers in MGC-803 cells and 35 node numbers in MKN-45 cells, suggesting that Sox2 might be a crucial downstream gene of Notum (Fig. 6f). The interaction between Notum and Sox2 were analyzed using String and mapped the Protein-Protein Interaction (PPI) network (Fig. 6g).

### Notum promotes GCSCs properties through upregulation of Sox2 by PI3K/AKT signaling pathway

**Fig. 7:**
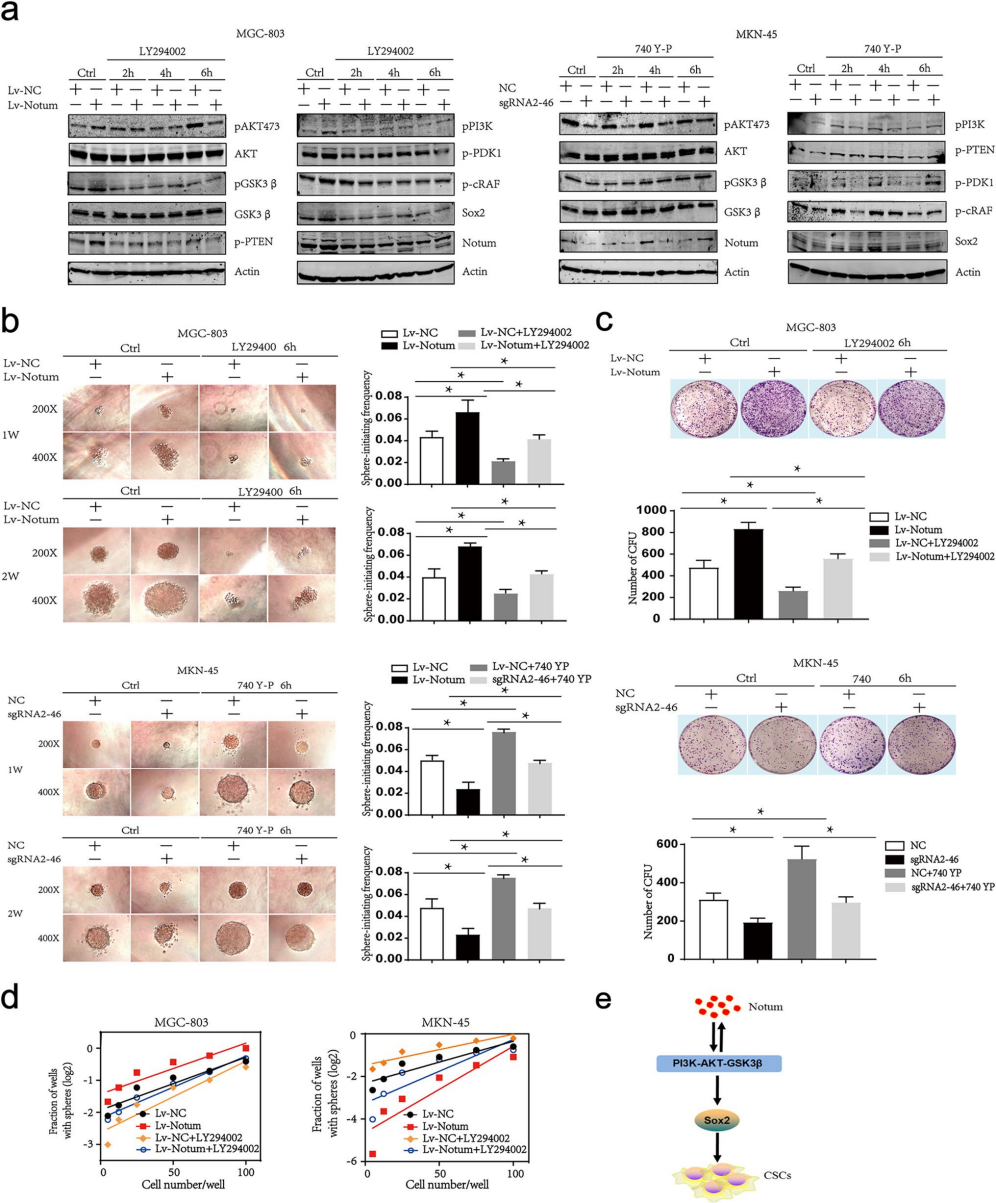
Notum enhances CSCs stemness through upregulation of Sox2 by PI3K/AKT signaling pathway. **a** Western blot analysis of the mutual effects between Notum andPI3K/AKT/Sox2 signaling pathway. **b**, **c**,** d** The ability of tumor sphere-initiating is accessed by single cell tumor sphere formation assays in vitro (b), colony formation assays (c) and limiting dilution assays in vitro (d). **e** A schematic diagram illustrating the proposed molecular mechanisms of Notum in GCSCs

Previous reports have established that PI3K/AKT signaling pathway is considered crucial for maintenance of cellular stemness, and Sox2 as its downstream target has also been identified. Therefore, we respectively treated Notum-overexpressing MGC-803 cells with PI3K/AKT signaling pathway inhibitor LY294002, and Notum silenced MKN-45 cells with activator 740-YP to inhibit and to activate the PI3K/AKT signaling pathway. As shown in Fig. 7a, LY294002 significantly decreased p-PI3K, p-AKT (Thr473) and p-GSK3β levels in Notum-overexpressing MGC-803 cells, and on the other hand, the expressions of p-PI3K, p-AKT (Thr473) and p-GSK3β were restored after 6 h 740-YP treatment following Notum knockdown in MKN-45 cells. Of note, Notum and PI3K/AKT signaling pathway might be the bidirectional regulation. That was to say, Notum might affect the activity of PI3K/AKT signaling pathway and was mediated by it at the same time. Moreover, we found that Sox2 was regulated by Notum and PI3K/AKT signaling pathway, but overexpression and knockdown of Sox2 had no effects on levels of Notum and PI3K/AKT associated proteins, suggesting that Sox2 might be a downstream target of Notum and PI3K/AKT signaling pathway (Fig. 7a and Additional file 1: Fig. S8D).

Next, we examined the role of PI3K/AKT/Sox2 signaling pathway in Notum-induced stemness. As illustrated in Fig. 7b, Notum ectopic expression increased the efficiency of the single cell tumor sphere formation and LY294002 stimulating reversed the ability of Notum-overexpressing MGC-803 cells to promote spheres formation in vitro. Meanwhile, silencing Sox2 markedly abrogated the sphere-forming ability of Notum-overexpressing MGC-803 cells, whereas Sox2 overexpression restored the tumor sphere formation inhibited by Notum knockdown (Additional file 1: Fig. S8C). Subsequently, the colony formation and limiting dilution analysis were performed to further demonstrate the role of Notum on stemness prosperities driven by Sox2 and PI3K/AKT signaling pathway (Fig. 7c and d and Additional file 1: Fig. S8A, S8B). Overall, our results indicated that the bidirectional regulation between Notum and PI3K/AKT signaling pathway resulted in upregulation of Sox2, therefore enhancing GC stem cell-like traits and triggering tumor progression in GC patients (Fig. 7e).

### Caffeine is a promising intervention for GC patients

**Fig. 8:**
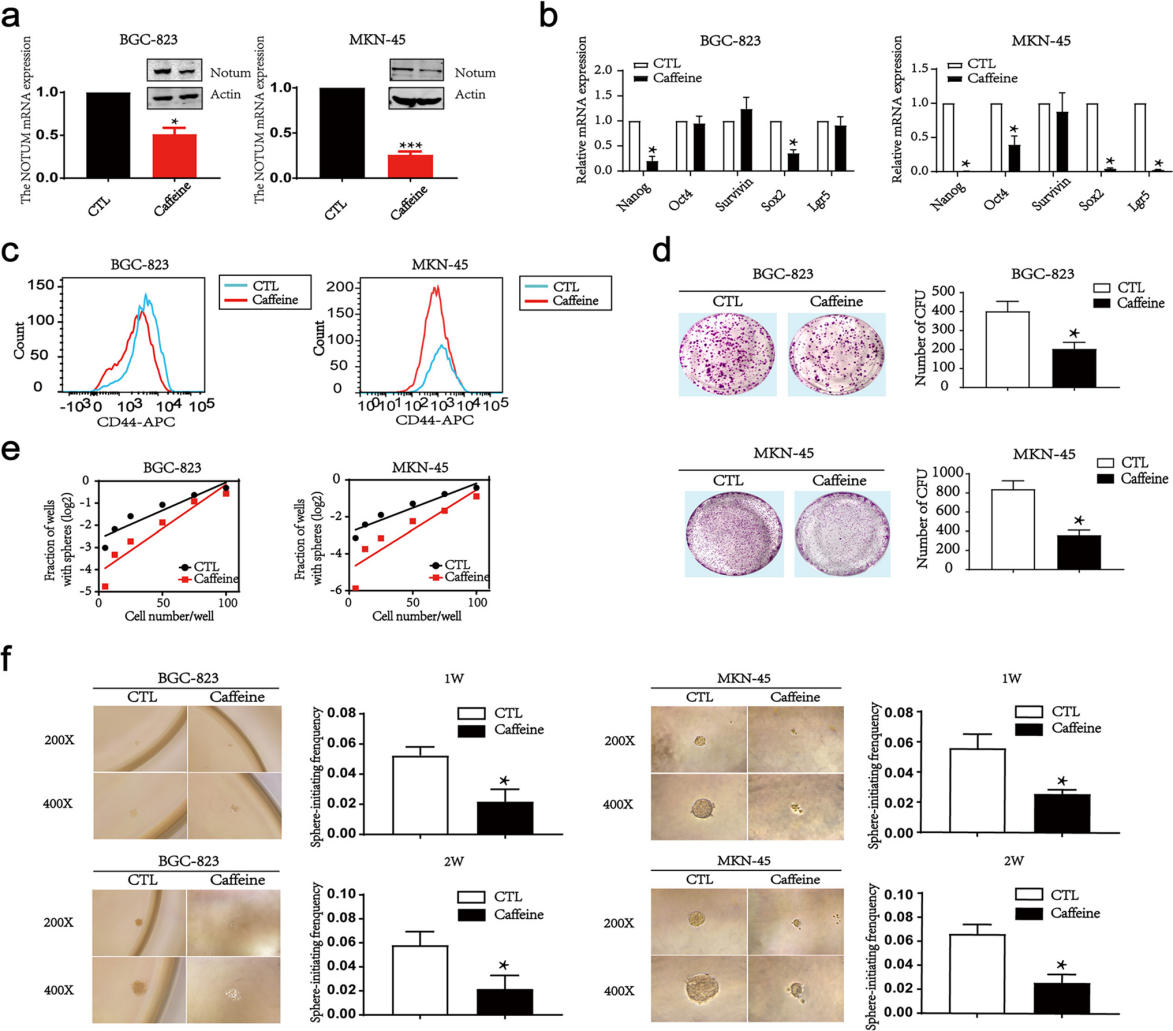
Caffeine inhibits Notum expression and tumor spheres formation in GC. **a** Notum levels are analyzed by RT-qPCR and western blot after Caffeine treatment. **b**, **c** The stemness genes and CD44 expressions are measured by RT-qPCR (b) and flow cytometry (c) in BGC-823 and MKN-45 cells treated with Caffeine. **d**, **e**, **f** The capacity of colony formation and tumor sphere-initiating are accessed by colony formation assays (d), limiting dilution assays (e) and tumor sphere formation assays in vitro (f) after Notum inhibition by Caffeine

To translate the bench results into clinical practice, four compounds reported as inhibitors of Notum were investigated. We found that Caffeine dramatically decreased the expression of Notum, suggesting the inhibitory effect of novel drug Caffeine in GC (Fig. 8a and Additional file 1: Fig. S9A). To this end, a series of functional experiments were conducted. As expected, Caffeine significantly decreased the expressions of stemness markers including Nanog, Sox2 and CD44 (Fig. 8b and c). We next examined the impact of Caffeine on cell viability and colony formation. As shown, Caffeine had no effect on cell viability at the time courses used, but a minor inhibitory effect on colony formation (Fig. 8d and Additional file 1: Fig. S9B). The limiting dilution assay and tumor spheres formation analysis further demonstrated that Caffeine attenuated the ability of tumor spheres formation in vitro (Fig. 8e and f). Mechanically, p-PI3K, p-AKT (Thr473), p-GSK3β and Sox2 levels were reduced after treating with Caffeine in GC cell lines, suggesting Caffeine might mediate the expression of Sox2 and the activity of PI3K/AKT signaling pathway (Additional file 1: Fig. S9C). Thus, Caffeine might be a novel and effective therapeutic agent for targeting GCSCs.

## Discussion

Increasing evidence has revealed that GCSCs are a key population of tumor cells with highly tumorigenic and chemoresistant abilities [25–28]. Conventional anticancer therapies such as chemotherapy, radiation, and immunotherapy have been used, which can only kill differentiated tumor cells but not GCSCs. Due to lack of the specific markers, it is still a considerable challenge to targeting GCSCs [29]. Exploiting the property of GCSCs resistant to chemotherapy, we generated highly malignant GC cell lines by treating 5-FU or Cisplatin in secondary tumor spheres. Our data showed that Cis-treated sphere cells may largely enrich the stem-like cells and exhibit the stemness properties much closer to GCSCs, including an enhanced sphere formation capacity, the GCSCs phenotypes (CD44^+^, Lgr5, Sox2 et al.) and a strong tumorigenicity [30–32]. Therefore, the enrichment and acquisition of large number of GCSCs with chemotherapy is a good method for studying GCSCs. Currently, several novel strategies have been suggested, including targeting GCSCs cell surface molecules, inhibiting GCSCs pathways, a combination of chemotherapy-associated apoptosis [33]. Our previous study found that ICG-001, a small molecular inhibitor of Wnt signaling pathway, significantly augmented the killing effect of chemotherapy drugs on stem-like cancer cells in GC [34]. Although great progresses have been made in the field of targeting GCSCs, master regulators of cellular genes and detail mechanisms remain largely unknown.

It is generally accepted that Notum is a glypican-specific phospholipase with a typical α/βhydrolase domain and first known deacylated extracellular protein enzyme [35].Emerging evidence have shown that Notum is overexpressed in some primary cancers and served as the biomarker for prognostic. The latest study found that Notum was up-regulated in hepatoblastoma and Notum knockdown distinctly weakened migration and invasion and tumor growth in vivo [36]. Our data indicated that Notum was aberrantly upregulated in GC, especially in early stage and stem like GC cells. Importantly, serum Notum possessed the highest diagnostic power in discriminating early stage GC patients and advanced patients suggesting that Notum could serve as a potential predictive marker for the diagnosis of early-stage GC.

Consistent with previous studies, Notum might participate in promoting stemness prosperities and tumorigenicity [19, 37]. Overexpression of Notum dramatically triggered a molecular profile of enhanced stemness and tumorigenicity, whereas genetic or pharmacologic inhibition of Notum markedly attenuated the efficiency of tumor sphere formation and the ability of tumorigenicity in vivo. Interestingly, the expressing location and secreting mode of Notum appeared to determine its regulatory function, and Notum functioned depending on the certain genes. As reported that Apc-mutant cells are enriched for Notum levels and inhibition of Notum abrogated the ability of Apc-mutant cells to expand and form intestinal adenomas. However, conditional medium from Apc-mutant cells that were high Notum-secreting clones actively inhibited the proliferation of surrounding wild-type crypt cells and drove their differentiation, thereby outcompeting crypt cells from the niche [38]. Combined with results of our data, we speculated that Notum might exert different functions through extracellular and intracellular pathways, and the feedback of Notum and Wnt proteins together regulated cell development and tumor growth. Our results elucidated that exogenous intervention of intracellular Notum level in GC cell lines leaded to change in abilities of tumor sphere formation and tumorigenicity. However, its extracellular functions remain unclear and further research are needed to be explored.

The abnormalities of PI3K/AKT signaling pathway and its roles in aberrant signaling cascades in human cancers have been validated, such as lung cancer, breast cancer, prostate cancer, colon cancer, gastric cancer and so on [39]. Further studies showed that the importance of PI3K/AKT signaling pathway in the maintenance of CSCs [40, 41]. In GC, the aberrantly activation of PI3K/AKT signaling pathway was able to contribute to the progression into highly malignant types characterized with enhanced abilities of metastasis, tumorigenicity and resistance to chemotherapy [42–44]. Here, we found that Notum increased efficiency of tumor sphere formation and ability of tumorigenicity through PI3K/AKT signaling pathway. Inhibition or activation of PI3K/AKT signaling pathway could recover the effects of Notum knockdown or overexpression on GC stemness. Of note, the relationship between Notum and PI3K/AKT activation might be bidirectional, but further studies needed to confirm this potential relationship. To define the mechanisms by which Notum modulates stemness, we evaluated known targets regulated by PI3K/AKT signaling pathway. From the results of RNA-Seq data, Sox2 expression change significantly after Notum overexpression and knockdown in GC, thus extremely attracting our attention. Past research had revealed that Sox2 exerts in oncogenesis, reprogramming, and diverse cancers, especially in caner stem cells [45–47]. As a key transcription factor, Sox2 protein stability, nuclear localization, and DNA regulation are determined by the PI3K/AKT/Sox2 signaling axis and directly modulate stemness, reprogramming, and cancer [48].Through functional experiments, we also identified that the stemness of GC was enforced by Sox2, as well as PI3K/AKT signaling pathway. Additionally, Notum knockdown or overexpression could impair or enhance stemness of GC cells by targeting Sox2.Therefore, we concluded that Notum mediated tumor sphere formation and tumorigenicity through upregulation of Sox2 by PI3K/AKT signaling pathway.

For a molecular target to be druggable, with the potential for translation to be the clinic, small-molecule Notum inhibitors as a potential therapeutic for GC have been explored, including LP-922,056, ABC99, Melatonin and Caffeine [21, 49, 50]. In the present study, we demonstrated that Caffeine could significantly reduce the level of Notum and inhibit the stemness of GC cells. Previous study reported that caffeine, a commonly consumed alkaloid, bonds the Wnt-decylase Notum and inhibits its activity [51]. And Caffeine could induce apoptosis and promote autophagy in GC, suggesting it’s the prospective treatment for GC [52, 53]. Our study found that Caffeine inhibited the plated colony formation and tumor sphere formation in suspension culture. In addition, Caffeine decreased the level of Sox2 and some key molecules in PI3K/AKT signaling pathway, suggesting Sox2 and PI3K/AKT signaling pathway might involve in regulation of Caffeine-mediating stemness prosperities and tumorigenicity. Besides, the results also elicited other mechanisms might involve in regulation of Caffeine on GC cells tumor sphere formation.

**Fig. 9:**
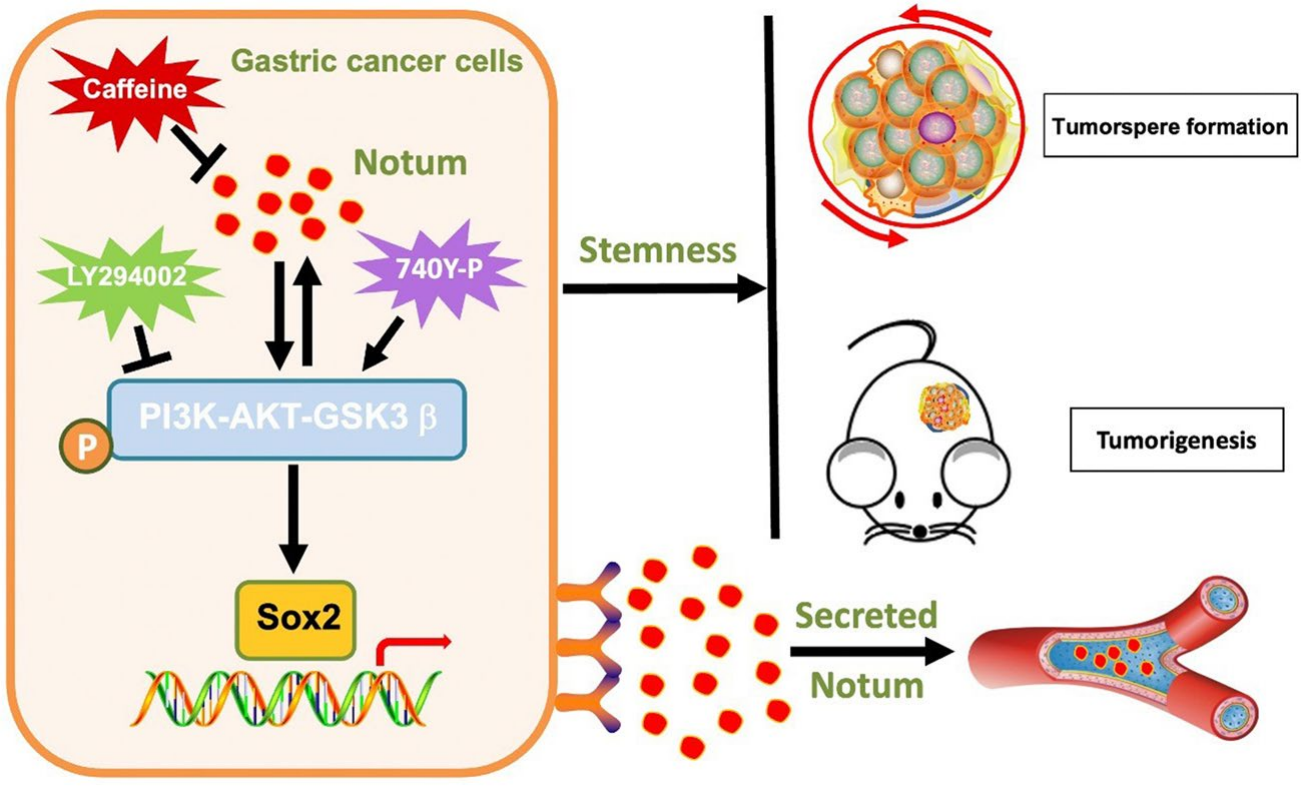
Schematic model for how Notum promotes GCSCs properties through upregulation of Sox2 by PI3K/AKT signaling pathway. In brief, Notum was identified as a novel key oncogene implicated in increasing efficiency of tumor sphere formation and ability of tumorigenicity through upregulation of Sox2 by PI3K/AKT signaling pathway. Moreover, Notum could be served as a potential predictive marker for the diagnosis of early-stage GC

In conclusion, we demonstrated for the first time that Notum upregulated the level of Sox2 byPI3K/AKT signaling pathway to promote tumor sphere formation and tumorigenicity in GC and found that Caffeine as Notum inhibitor could effectively abrogated GC spheroids formation but not affect cell growth. Moreover, serum Notum could serve as a potential predictive marker for the diagnosis of early-stage GC. These results provide the strong evidence that targeting Notum might be an appealing approach to predict the early-stage GC and tremendously retard the progression of GC patients (Fig. 9).

## Supplementary Information

Below is the link to the electronic supplementary material.

ESM 1 (DOCX 3.54 MB)

## Data Availability

Not applicable.

## Abbreviations

GC: Gastric cancer
GCSCs: Gastric cancer stem cells
GPCs: Glypicans
CRC: Colorectal cancer
RT-PCR: Real-time PCR
UICC: Union Against Cancer
KEGG: Kyoto Encyclopedia of Genes and Genomes
GO: Gene Ontology
DEGs: Differentially expressed genes
GEO: Gene Expression Omnibus
TCGA: The Cancer Genome Atlas
WGCNA: Weighted correlation network analysis
PPI: Protein-protein interaction
Cis: Cisplatin
GC Os: Gastric cancer organoids
FCA: Flow cytometric analysis
hES: Human embryonic stem
