# Supplementary material for: Notum enhances gastric cancer stem-like cell properties through upregulation of Sox2 by PI3K/AKT signaling pathway
Source: Cell Oncol (Dordr). 2023 Sep 26;47(2):463–80. doi: 10.1007/s13402-023-00875-w (PMC11090966; doi:10.1007/s13402-023-00875-w)
Supplement: Supplementary file 1 — (DOCX 3.54 MB) [file 13402_2023_875_MOESM1_ESM.docx]

**Supplementary Information**

**Notum enhances gastric cancer stem-like cell properties through upregulation of Sox2 by PI3K/AKT signaling pathway**

1. **Additional file 1: FigureS1-S9.**
2. **Additional file 2:TableS1-S6.**

**Additional file 1: Figure S1**

**
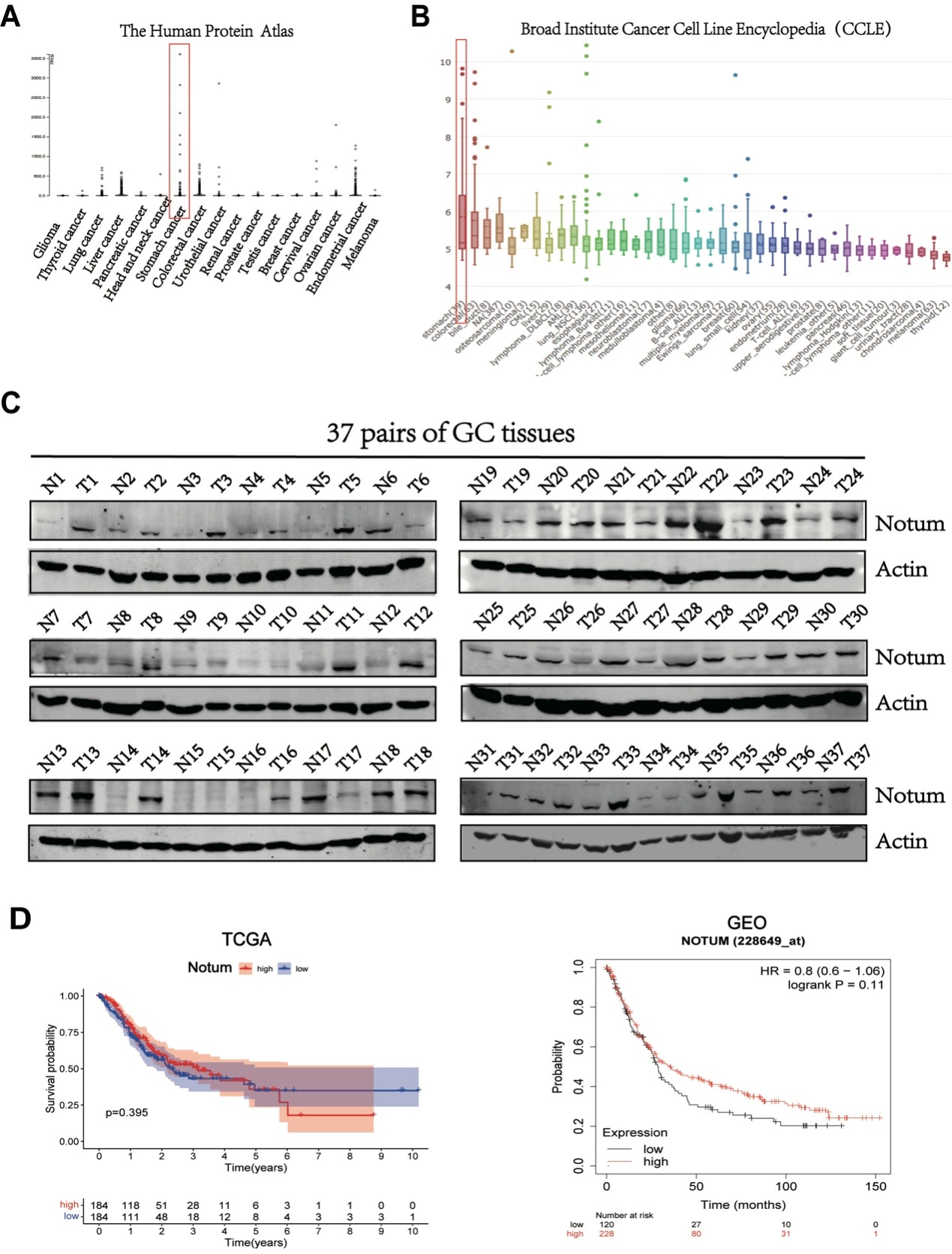
**

**Figure S1.** Notum is upregulated in GC patients. **A** and **B** Notum levels in different types of cancers from The Human Protein Atlas (A) and CCLE database (B). **C** Representative western blot analysis of 37 pairs of GC tissues and adjacent tissues. **D** Kaplan-Meier (K-M) analysis of overall survival of GC patients according to Notum levels (data from the TCGA and GEO datasets).

**Additional file 1: Figure S2**

**
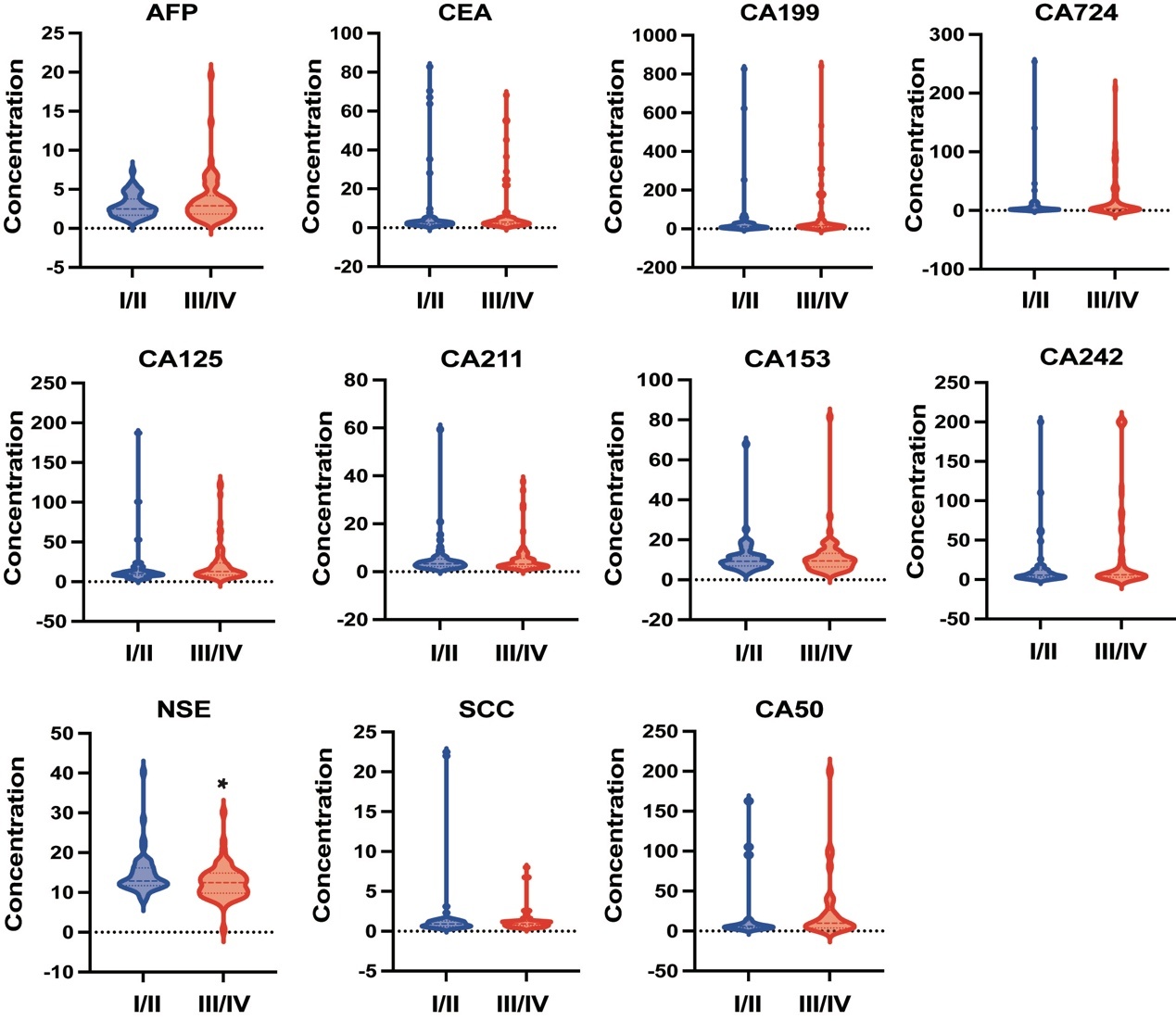
**

**Figure S2.** Levels of serum tumor markers in different stages of GC patients, including AFP, CEA, CA199, CA724, CA125, CA211, CA153, CA242, CA153, NSE, SCC and CA50.AFP: alpha fetoprotein, CEA: carcinoembryonic antigen, CA: cancer antigen. NSE: neuron-specific-enolase, SCC: squamous cell carcinoma. * P<0.05.

**Additional file 1: Figure S3**


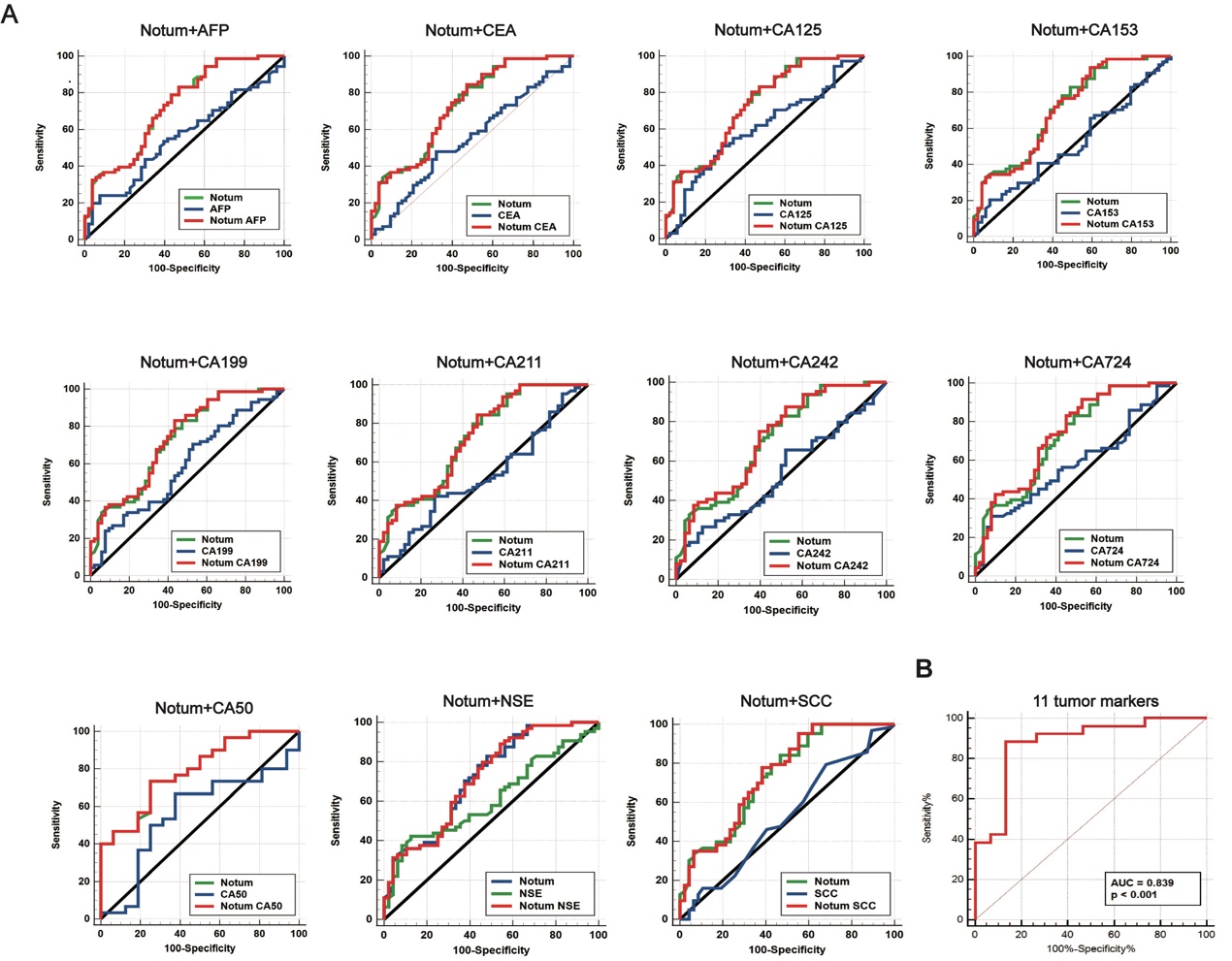


**Figure S3.** Receiver operating characteristic curves for the combination of serum Notum and other tumor markers to discriminate early stage of gastric cancer patients from advanced patients. **A** The diagnostic capacity of combination of Notum with each tumor marker. **B** ROC for the combination of 11 tumor markers.

**Additional file 1: Figure S4**


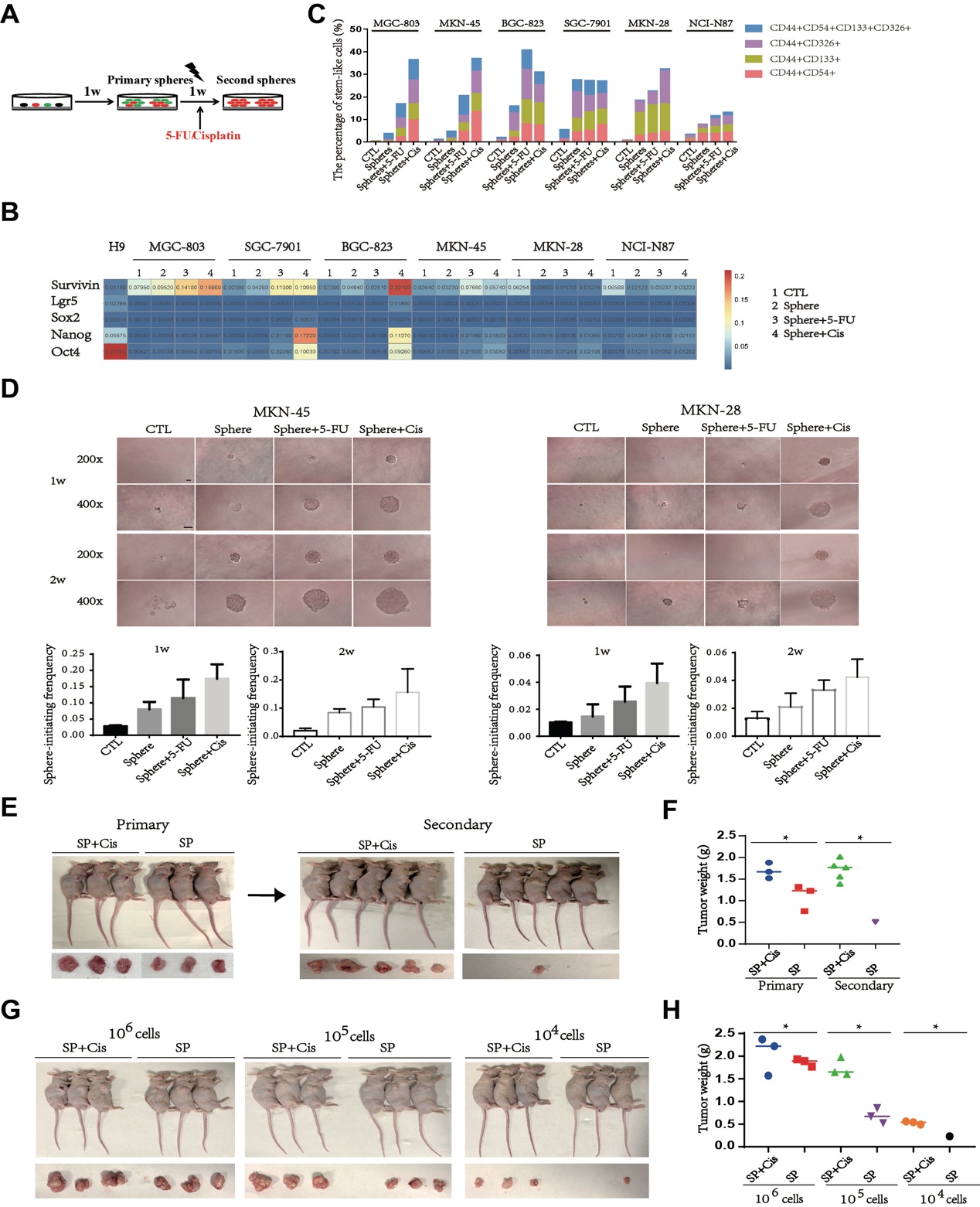


**Figure S4.** Cisplatin selectively enriches for GCSCs. **A** Schematic showing the cultivation approach, as described in the Material and Methods. **B** The relative levels of mRNA transcripts for stemness genes, including Survivin, Lgr5, Sox2, Nanog and Oct4. **C** Flow cytometric analysis for CD44, CD54, CD133 and CD326 cell surface expression in 6 GC cell lines cultured as spheres (GCSCs). **D** Sphere formation assay for MKN-45 and MKN-28 GC cell lines. Images are representative of tumor spheres taken at first week and second week after plating (Upper). Graphs show the percentage of the total number of wells for forming spheres/total number of wells for seeding cells (Sphere-initiating frequency). **E** and **F** The secondary xenograft transplantation experiment *in vivo* is applied to detect the tumorigenesis of MKN-45 tumor spheres after treatment with Cisplatin (E) and the statistical analysis of tumor weights is performed (F). **G** and **H** Representative images (G) and a statistical scattergram (H) showing the increased proportion of nude mice subcutaneously inoculated with serially diluted (10^6,^ 10^5^ and 10^4^) tumor spheres generated by MKN-45 cell lines after treatment with Cis or not.

**Additional file 1: Figure S5**
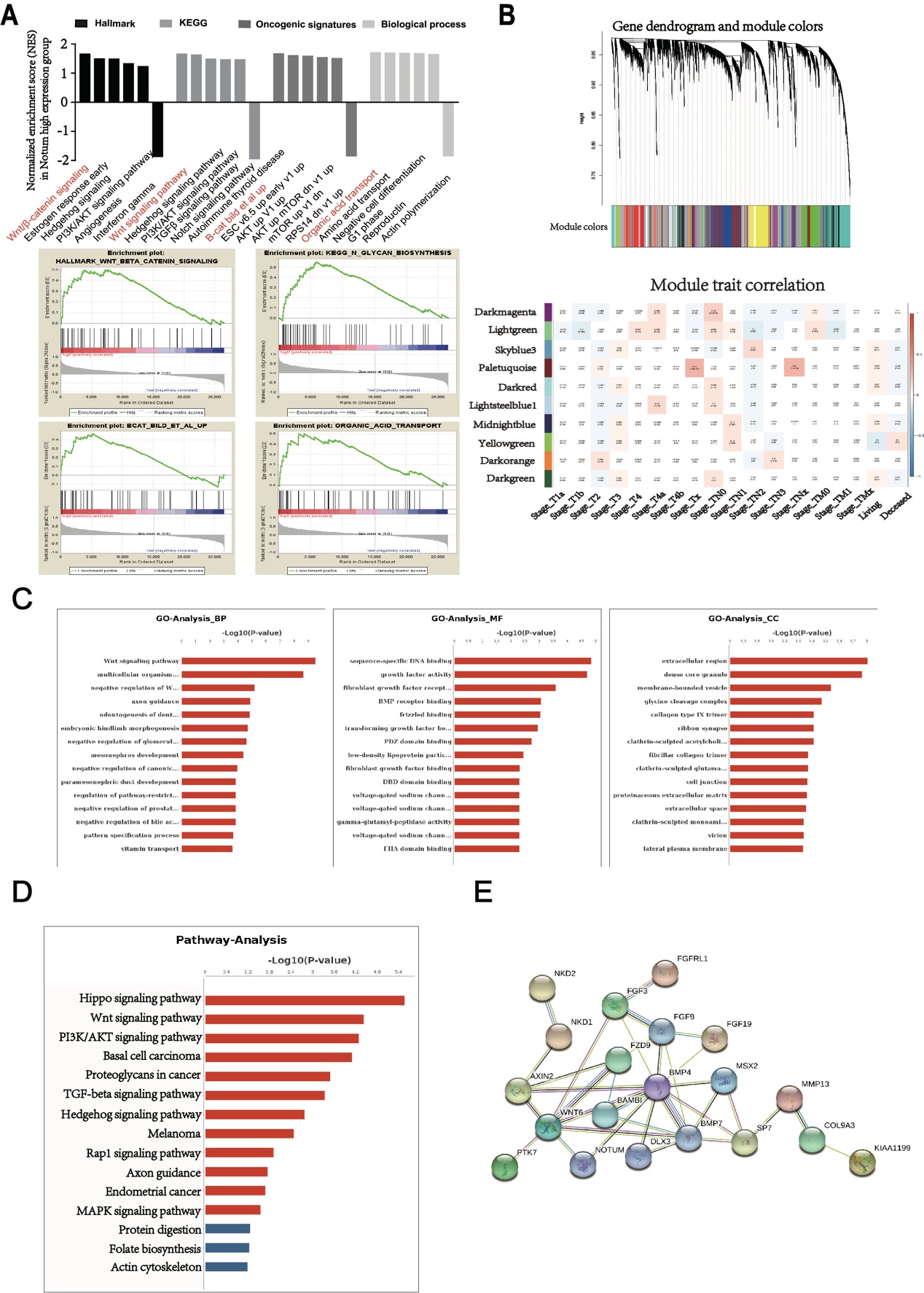


**Figure S5.** Bioinformatics analysis predicts the involvement of Notum in GCSCs development. **A** Gene Set Enrichment Analysis (GSEA) is applied to calculate the normalized enrichment score (NES) usingthe general gene set categories. The ones with the highest scores are highlighted and representative images are shown (lower). **B** Gene functions are predicted by Weighted correlation network analysis (WGCNA) from TCGA dataset. **C** and **D** The results of GO (C) and KEGG (D) analysis show the involvement of PI3K/AKT signaling pathway in cell biological function mediated by Notum. **E** String analysis of the relationship of Notum with other genes.

**Additional file 1: Figure S6**


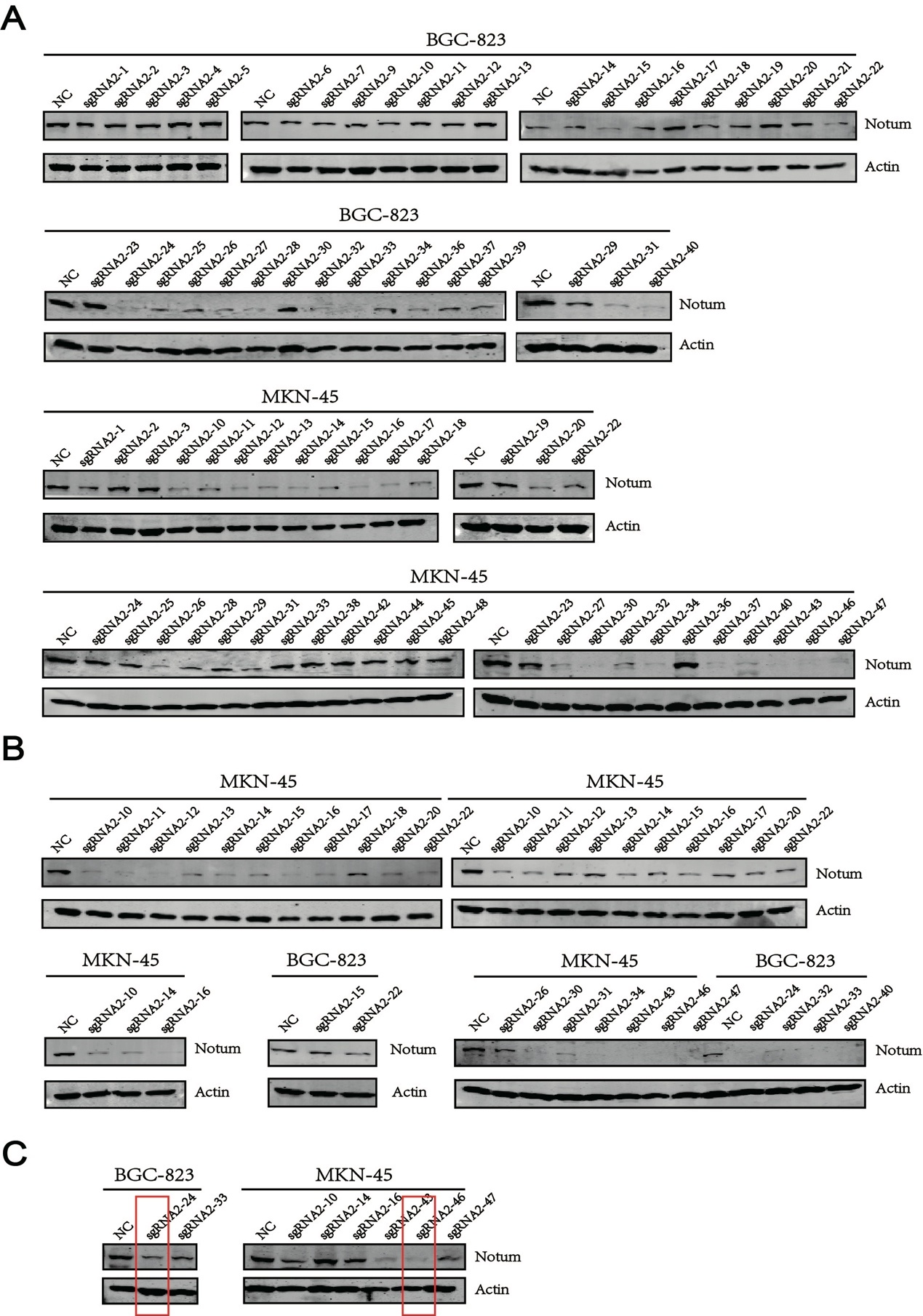


**Figure S6.** Silencing Notum with Crispr/Ca9 system. **A** Different silencing efficiency of colonies with Crispr/Cas system on Notum levels. **B** Further experiments are performed to confirm the silencing efficiency. **C** The colonies of sgRNA-24 in BGC-823 cells and sgRNA-46 in MKN-45 cells have the much higher and more stable silencing efficiency.

**Additional file 1: Figure S7**
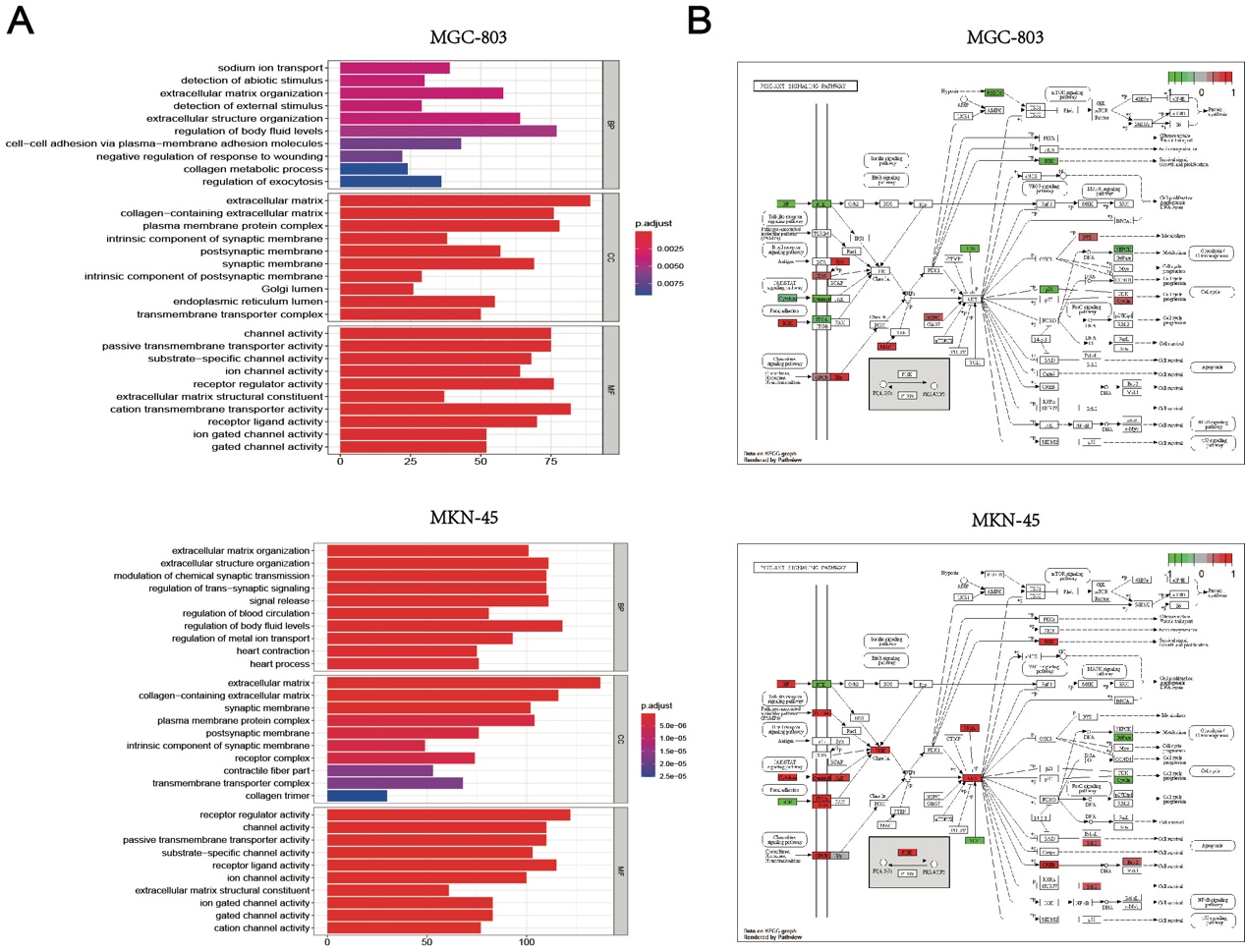


**Figure S7.**GO and KEGG analysis of the RNA-seq data in GC cell lines. **A** statistical histogram of the top 10 in biological process (BP), cellular component (CC) and molecular function (MP), respectively. **B** The involvement of PI3K/AKT signaling pathway in MGC-803 and MKN-45 cell lines analyzed by KEGG pathway database.

**Additional file 1: Figure S8**
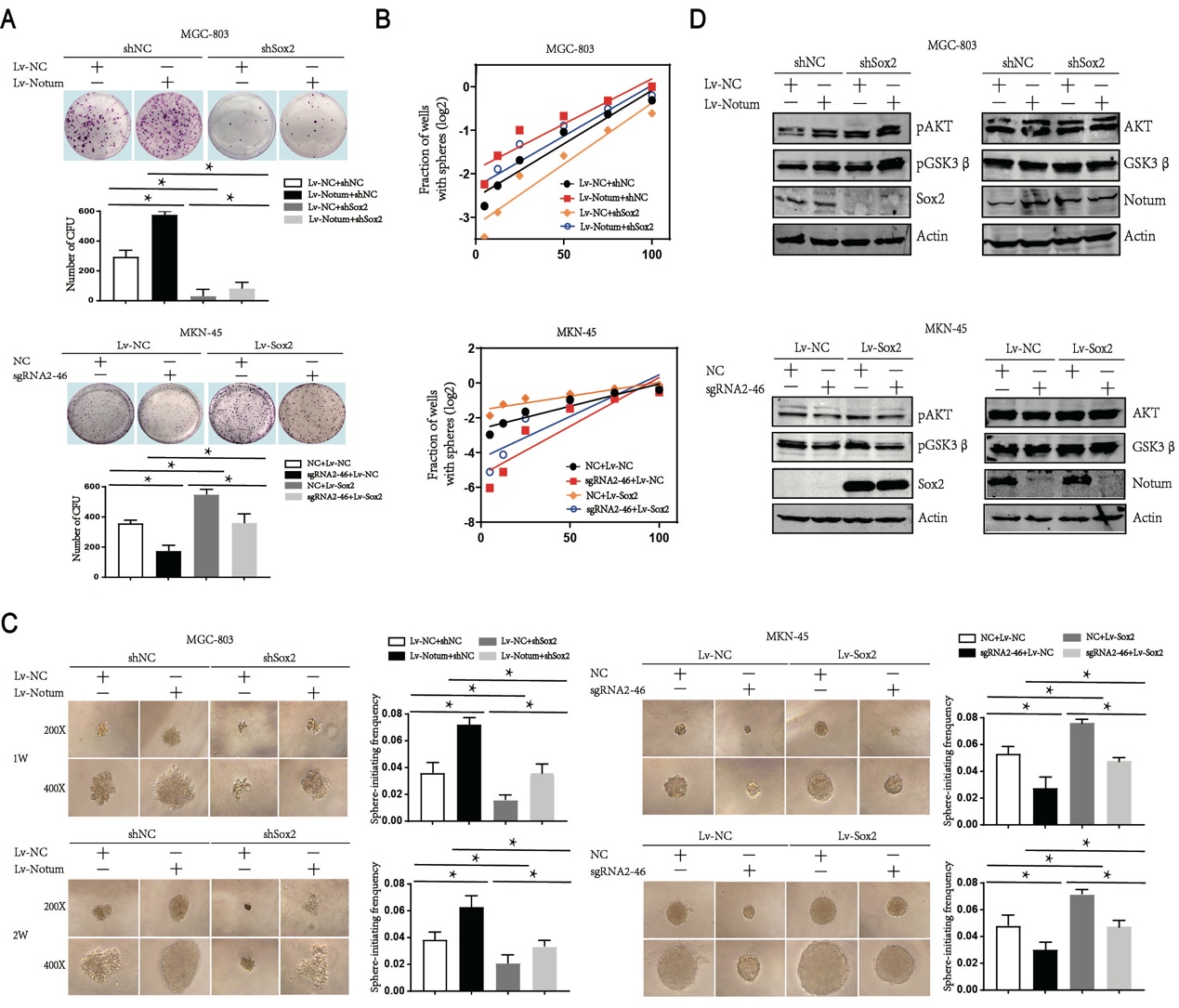


**Figure S8.** Notum exerts tumor sphere-initiating ability through targeting Sox2. **A** Representative image (upper) and a statistical histogram (lower) of colonies in Notum-expressing MGC-803 cells transduced with shSox2 vectors and Notum-silencing MKN-45 cells transduced with Lv-Sox2 vectors. **B** and **C** The limiting dilution (B) and single cell tumor sphere formation assays *in vitro* (C) are used to test the effects of Sox2 overexpression and knockdown on frequency of sphere-initiating cells. **D** Western blot analysis of the mutual effects between Notum and Sox2.

**Additional file 1: Figure S9**

**
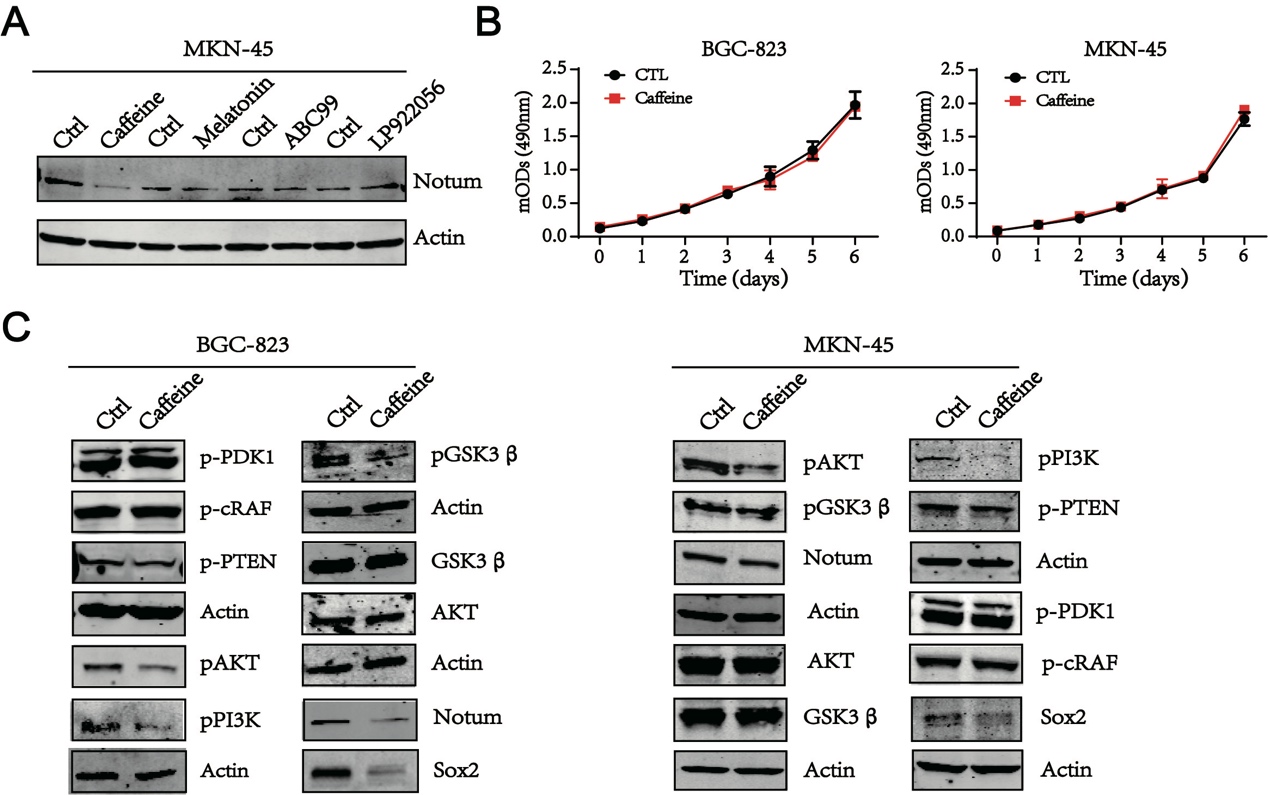
Figure S9.** Caffeine attenuates PI3K/AKT/Sox2 signaling pathway. **A** Inhibitory efficiency of four compounds reported as inhibitors of Notum are confirmed by western blot in MKN-45 cells. **B** MTT analysis of Notum inhibition by Caffeine on cell viability. **C** Effects of Caffeine on PI3K/AKT/Sox2 signaling pathway analyzed by western blot in BGC-823 and MKN-45 cells.

**Additional file 2:Table S1**

TableS1.Summary of patient characteristics (Serum)

| **Clinicopathological**  **feature** | **Number of**  **cases** | **Expression of Notum**  **(mean ± SEM)** | **P value** |
| --- | --- | --- | --- |
| **Gender** |  |  |  |
| Male | 95 | 2.7916 ±1.86090 | 0.720 |
| Female | 37 | 2.6689± 1.46545 |  |
| **Age(years)** |  |  |  |
| ≥60 | 96 | 2.8865 ± 1.84598 | 0.168 |
| <60 | 36 | 2.4125 ± 1.45036 |  |
| **Lymphatic invasion** |  |  |  |
| Yes | 21 | 1.8757 ± 1.65250 | 0.011* |
| No | 111 | 2.9240 ± 1.72997 |  |
| **TNM stage** |  |  |  |
| І/Ⅱ | 56 | 3.5712± 1.82501 | 0.000*** |
| Ⅲ/Ⅳ | 76 | 2.1574 ± 1.44036 |  |
| **Tumor location** |  |  |  |
| Cardiac part | 14 | 1.590±1.070 |  |
| Fundus of stomach | 3 | 1.117±0.448 |  |
| Body of stomach | 13 | 1.711±1.651 |  |
| Pyloric part | 28 | 1.419±0.874 |  |
| Missing | 74 | 1.436±1.146 |  |
| **Differentiation** |  |  |  |
| Well | 2 | 4.1125 ± 2.17860 |  |
| Moderate | 5 | 1.5838 ± 1.10556 |  |
| Poor | 6 | 1.3052 ± 1.11359 | 0.048^1^* |
| Missing | 119 | 2.8569 ± 1.75394 | 0.033^2^* |

1.Well vs Poor, 2. Poor vs Missing, * P<0.05, ***P<0.001

**Additional file 2:Table S2**

TableS2.Summary of patient characteristics (IHC)

| **Clinicopathological**  **feature** | **Number of**  **cases** | **Expression of Notum**  **(mean ± SEM)** | **P value** |
| --- | --- | --- | --- |
| **Gender** |  |  |  |
| Male | 45 | 150.7036 ± 33.96114 | 0.710 |
| Female | 18 | 155.3083 ± 29.69038 |  |
| **Age(years)** |  |  |  |
| ≥60 | 40 | 155.8269 ± 19.85623 | 0.374 |
| <60 | 23 | 145.5755 ± 47.10565 |  |
| **Lymphatic invasion** |  |  |  |
| Yes | 34 | 155.4503 ± 34.63635 | 0.363 |
| No | 29 | 144.5333 ± 26.97073 |  |
| **PTNM stage** |  |  |  |
| І/Ⅱ | 25 | 177.6234± 44.82744 | 0.0341* |
| Ⅲ/Ⅳ | 38 | 119.2124 ± 20.93751 |  |
| **Differentiation** |  |  |  |
| Well | 4 | 163.8665 ± 21.78601 |  |
| Moderate | 36 | 156.2471 ± 11.00556 |  |
| Poor | 23 | 147.6586 ± 12.13590 |  |

* P<0.05

**Additional file 2:Table S3**

TableS3.Summary of patient characteristics

| **Clinicopathological**  **feature** | **Number of**  **cases** | **Expression of Notum**  **(mean ± SEM)** | **P value** |
| --- | --- | --- | --- |
| **Gender** |  |  |  |
| Male | 27 | 0.0025 ± 0.0075 | 0.421 |
| Female | 18 | 0.0010 ± 0.0023 |  |
| **Age(years)** |  |  |  |
| ≥60 | 30 | 0.0018 ± 0.0067 | 0.921 |
| <60 | 15 | 0.0020 ± 0.0043 |  |
| **Lymphatic invasion** |  |  |  |
| Yes | 28 | 0.0008 ± 0.0027 | 0.116 |
| No | 17 | 0.0037 ± 0.0089 |  |
| **TNM stage** |  |  |  |
| І/Ⅱ | 18 | 0.0043 ± 0.0088 | 0.038* |
| Ⅲ/Ⅳ | 27 | 0.0006 ± 0.0018 |  |
| **Tumor location** |  |  |  |
| Cardiac part | 3 | 0.0002 ± 0.0001 |  |
| Fundus of stomach | 1 | 0.0001 ± 0.0000 |  |
| Body of stomach | 21 | 0.0008 ± 0.0020 |  |
| Pyloric part | 14 | 0.0013 ± 0.0038 |  |
| Missing | 6 | 0.0081 ± 0.0143 |  |
| **Differentiation** |  |  |  |
| Well | 1 | 0.0000 ± 0.0000 |  |
| Moderate | 8 | 0.0002 ± 0.0003 |  |
| Poor | 15 | 0.0004 ± 0.0005 |  |
| Missing | 21 | 0.0037 ± 0.0085 |  |

* P<0.05

**Additional file 2:Table S4**

TableS4.Primers used in study

| **Name** | **Forward sequence (5’-3’)** | **Reverse sequence (5’-3’)** |
| --- | --- | --- |
| ACTIN | TGAAGTACCCCATCGAGCACGGCA | GATAGCACAGCCTGGATAGCAACG |
| Survivin | CTTGGCCCAGTGTTTCTTCT | TCTCCGCAGTTTCCTCAAAT |
| cyclinD1 | CTCGGTGTCCTACTTCA | TCCTCGCACTTCTGTTC |
| Lgr5 | TGTGCATTTGGAGTGTGTGA | CACGTTCATCTTGAGCCTGA |
| Notum | ACTCCAGATACGACACCATGC | CATCACTGGAGCAGTAGGGG |
| Nanog | GGTTCCAGAACCAGAGAATGAAA | GTTGCTCCACATTGGAAGGTT |
| Oct4 | GGTATTCAGCCAAACGACCAT | ACGAGGGTTTCTGCTTTGCA |
| Sox2 | CTCGCCCACCTACAGCAT | GACTTGACCACCGAACCC |

**Additional file 2:Table S5**

TableS5.Antibodies used in study

| **Antibody** | **Source** | **Country** |
| --- | --- | --- |
| Notum | Abcam | USA |
| Sox2 | Abcam | USA |
| p-PI3K | Cell Signaling Technology | USA |
| p-GSK3β | Cell Signaling Technology | USA |
| GSK3β | Cell Signaling Technology | USA |
| p-AKT (Ser473) | Cell Signaling Technology | USA |
| AKT | Cell Signaling Technology | USA |
| p-AKT (Thr308) | Cell Signaling Technology | USA |
| p-PTEN | Cell Signaling Technology | USA |
| p-cRAF | Cell Signaling Technology | USA |
| p-PDK1 | Cell Signaling Technology | USA |
| PI3K-β | Cell Signaling Technology | USA |
| PI3K-γ | Cell Signaling Technology | USA |
| IRDye 800CW | LI-COR Biosciences | USA |
| IRDye 680CW | LI-COR Biosciences | USA |
| Actin | Cell Signaling Technology | USA |

**Additional file 2:Table S6**

Table S6.Diagnostic efficiencies of serum Notum and tumor markers in early-stage gastric cancer

|  | AUC | Sensitivity | Specificity | Youden index J | Significance level P(Area) |
| --- | --- | --- | --- | --- | --- |
| Notum | 0.728 | 82.89 | 51.79 | 0.3468 | <0.0001*** |
| AFP | 0.556 | 23.94 | 92.45 | 0.164 | 0.2796 |
| CEA | 0.548 | 47.89 | 67.92 | 0.1581 | 0.362 |
| CA125 | 0.601 | 50.7 | 71.7 | 0.224 | 0.0498* |
| CA153 | 0.516 | 20.31 | 91.84 | 0.1215 | 0.7762 |
| CA199 | 0.588 | 70.42 | 47.17 | 0.1759 | 0.0902 |
| CA211 | 0.525 | 42.19 | 73.47 | 0.1566 | 0.6518 |
| CA242 | 0.716 | 87.5 | 47.92 | 0.3542 | <0.0001*** |
| CA724 | 0.581 | 30.99 | 92.16 | 0.2314 | 0.1188 |
| CA50 | 0.56 | 66.67 | 62.5 | 0.2917 | 0.5145 |
| NSE | 0.611 | 42.19 | 87.5 | 0.2969 | 0.0381* |
| SCC | 0.527 | 79.37 | 31.91 | 0.1128 | 0.6396 |
| Notum+AFP | 0.73 | 83.1 | 52.8 | 0.3593 | <0.0001*** |
| Notum+CEA | 0.548 | 47.89 | 67.92 | 0.15810 | 0.362 |
| Notum+CA125 | 0.601 | 50.7 | 71.7 | 0.224 | 0.0498* |
| Notum+CA153 | 0.516 | 20.31 | 91.84 | 0.1215 | 0.7762 |
| Notum+CA199 | 0.588 | 70.45 | 47.17 | 0.1759 | 0.0902 |
| Notum+CA211 | 0.525 | 42.19 | 73.47 | 0.1566 | 0.6518 |
| Notum+CA242 | 0.535 | 26.56 | 87.5 | 0.1406 | 0.5188 |
| Notum+CA724 | 0.581 | 30.99 | 92.16 | 0.2314 | 0.1188 |
| Notum+CA50 | 0.56 | 26.56 | 87.5 | 0.1406 | 0.5188 |
| Notum+NSE | 0.611 | 42.19 | 87.5 | 0.2969 | 0.0381* |
| Notum+SCC | 0.527 | 79.47 | 31.91 | 0.1128 | 0.6396 |
| 11 tumor markers | 0.839 | 80.00 | 88.46 | 0.4092 | <0.0001*** |
| Combination | 0.91 | 88.46 | 86.67 | 0.7513 | <0.0001*** |

* P<0.05, ***P<0.001
